# Bioenergy Cropping Reduces the Spatiotemporal Scaling of Soil Bacterial Biodiversity

**DOI:** 10.1002/advs.202518964

**Published:** 2026-03-17

**Authors:** Zhencheng Ye, Jialiang Kuang, Colin T. Bates, Arthur Escalas, Daliang Ning, Liyou Wu, Suo Liu, Sihang Deng, Jiesi Lei, Xiangwen Chen, Jennifer Pett‐Ridge, Malay Saha, Lauren Hale, Gangsheng Wang, Renmao Tian, Ying Fu, Yu Tang, Mary Firestone, Jizhong Zhou, Yunfeng Yang

**Affiliations:** ^1^ State Key Joint Laboratory of Environment Simulation and Pollution Control School of Environment Tsinghua University Beijing China; ^2^ Hainan Institute of National Park Haikou China; ^3^ The Institute for Environmental Genomics University of Oklahoma Norman Oklahoma USA; ^4^ College of Environment and Energy South China University of Technology Guangzhou China; ^5^ MARBEC University of Montpellier Montpellier France; ^6^ Physical and Life Sciences Directorate Lawrence Livermore National Laboratory Livermore CA USA; ^7^ Life & Environmental Sciences Department University of California Merced Merced CA USA; ^8^ Innovative Genomics Institute University of California Berkeley Berkeley CA USA; ^9^ Noble Research Institute Ardmore Oklahoma USA; ^10^ US Department of Agriculture (USDA) Agricultural Research Service (ARS) Water Management Research Parlier CA USA; ^11^ State Key Laboratory of Water Resources Engineering and Management and Institute for Water‐Carbon Cycles and Carbon Neutrality Wuhan University Wuhan China; ^12^ Institute of Environment and Ecology Tsinghua Shenzhen International Graduate School Tsinghua University Shenzhen China; ^13^ Department of Environmental Science Policy and Management University of California Berkeley California USA; ^14^ Earth and Environmental Sciences Area Lawrence Berkeley National Laboratory Berkeley California USA; ^15^ School of Biological Sciences University of Oklahoma Norman Oklahoma USA; ^16^ School of Civil Engineering and Environmental Sciences University of Oklahoma Norman Oklahoma USA; ^17^ School of Computer Sciences University of Oklahoma Norman Oklahoma USA

**Keywords:** bacterial diversity, bioenergy cropping, spatial scaling, species‐time‐area relationship, temporal scaling

## Abstract

Widespread bioenergy cropping can transform landscapes, strongly affecting biodiversity. However, the impact of bioenergy cropping on the spatiotemporal scaling of soil biodiversity remains virtually unknown, despite its profound implications for the functioning of the ecological community. Here, we investigated how bioenergy cropping influenced the spatiotemporal scaling of soil bacterial biodiversity in marginal soils (sandy loam and clay loam soils) in Oklahoma, USA. We detected strong, significant species‐time‐area relationships (STARs) and phylogenetic‐time‐area relationships (PTARs) in bacterial communities and their lineages, suggesting that STARs and PTARs exist in microbial ecology within the studied system. Also, spatiotemporal scaling rates (the slopes of STAR and PTAR models) varied substantially among bacterial lineages and were positively correlated with their 16S rRNA gene copy numbers, a genomic trait indicative of microbial growth potentials. Strikingly, bioenergy cropping significantly reduced spatiotemporal scaling rates by 6.8%‐14.1%, with a more pronounced reduction observed in sandy loam soils, where those rates were significantly lower than in clay loam soils. The heterogeneity of soil phosphorus and carbon resulted in variations in bacterial spatiotemporal scaling rates. Collectively, our findings suggest that bioenergy cropping may alleviate rapid shifts in soil biodiversity across space and time, thereby stabilizing soil biodiversity and supporting its role as part of sustainable land management and climate mitigation strategies.

## Graphical abstract

Consistent with patterns observed in plant and animal communities, soil bacterial communities exhibit significant species–time–area and phylogenetic–time–area relationships independent of nested structure. Bioenergy cropping significantly reduces the spatiotemporal scaling rates, particularly in sandy loam soils. The results suggest that bioenergy cropping stabilizes soil biodiversity, supporting sustainable land management and climate mitigation strategies.

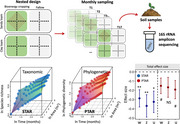

## 1 Introduction

A fundamental goal in ecology is to understand how biodiversity is generated and maintained across space and time [1, 2]. Such understanding is key to uncovering the underlying mechanisms shaping biodiversity, advancing ecological theories, and guiding biodiversity conservation efforts [3]. Among the well‐established spatial and temporal biodiversity patterns in ecology, the species‐area (SARs) and species‐time (STRs) relationships stand out, elucidating the increase in observed species richness as sampled spatial and temporal scales increased [4, 5], with their slopes in a log‐log space referred to as the scaling rates [6]. SARs and STRs have been extensively documented in macro‐ and micro‐communities across terrestrial and aquatic ecosystems [7, 8, 9, 10], with soil heterogeneity, dispersal limitation, and the Passive Sampling Hypothesis being the potential drivers [11]; thus, SARs and STRs may represent universal laws in ecology [7, 12].

Over two decades ago, it was proposed that SARs and STRs should not be considered separate phenomena. Instead, they form two dimensions of a unified model, i.e., species–time–area relationships (STARs) [1, 6]. The STAR model outperforms the simple single variable models where area and time are treated as independent variables [6], providing more accurate estimates of regional and local biodiversity, extinction rates [13], and species hotspots [14]. Furthermore, STAR quantifies time–space coupling rather than assuming independence and provides a quantitative metric of time–space equivalence. Also, the phylogenetic analog of STARs, known as the phylogenetic–time–area relationships (PTARs), are essential for defining conservation areas and periods that effectively preserve evolutionary history [15, 16]. Previous studies have demonstrated STAR and PTAR patterns in plants and animals [6, 17, 18]. However, STAR has been examined in only a single study of microbial ecology, but spatiotemporal interactions were not considered [19]. To the best of our knowledge, there was no such study on microbial biodiversity in nature, primarily due to the lack of time series observations under spatially nested sampling schemes.

Among the various types of land‐use changes that shape diversity distributions, the cultivation of bioenergy crops has attracted significant attention in recent years due to their potential to alleviate resource demands while enhancing carbon storage [20, 21]. Thus, large‐scale bioenergy cropping initiatives have been implemented on marginal lands unsuitable for food agriculture in numerous regions globally [22]. In general, bioenergy cropping can promote soil nutrient levels [23], which could support more microbial species by providing more energy [24] and generating more niches [25]. Therefore, it is expected that bioenergy cropping will increase the spatiotemporal scaling rates of soil microbial biodiversity. However, bioenergy cropping may also lead to a loss of soil heterogeneity due to intensive monoculture [26], which could act as a strong filtering factor against existing microbial species and decrease microbial diversity by diminishing niche availability. Therefore, bioenergy cropping could decrease the spatiotemporal scaling rates of soil microbial biodiversity. Furthermore, these opposing effects could be intertwined, resulting in no discernible change in the spatiotemporal scaling rates of biodiversity.

To determine whether and how bioenergy cropping affects the spatiotemporal scaling of soil microbial biodiversity, we examined both taxonomic and phylogenetic spatiotemporal patterns of soil bacterial communities in two sites with marginal soils (sandy loam and clay loam soils), with fallow and switchgrass (*Panicum virgatum* L.) cropping, over 17 months in Oklahoma, USA. Switchgrass is a tall, perennial, deep‐rooted grass native to the Central North American Plains [27]. It is a well‐studied bioenergy crop considered suitable for large‐scale cultivation in the United States, and it is projected to grow well in many regions globally, with or without irrigation [22]. It exhibits rapid growth and maintains high productivity even on low‐quality soils that are unsuitable for conventional row‐crop agriculture, typically with little to no additional inputs [28]. Switchgrass typically reaches full maturity within about two to three years [27], during which it establishes a robust structure and develops a deep root system. It is also highly drought‐tolerant [29] and can reduce topsoil erosion owing to its high root biomass, which increases the surface area for exudation [22]. Therefore, the effects of switchgrass cropping can already be observed within 17 months. Our main objectives were to answer the following questions: (i) whether STARs and PTARs exist in soil bacterial communities and if they are universally applicable across different bacterial lineages, (ii) how bioenergy cropping influences bacterial STARs and PTARs, and (iii) what are the underlying mechanisms. We hypothesize that (i) STARs and PTARs exist in bacterial communities, (ii) bioenergy cropping reduces the spatiotemporal scaling rates of both taxonomic and phylogenetic diversity, and (iii) the reduction is due to decreased soil heterogeneity. As expected, our results indicated that soil bacterial communities and their lineages exhibited strong, significant STAR and PTAR patterns, and bioenergy cropping significantly reduced the taxonomic and phylogenetic spatiotemporal scaling rates of soil bacterial diversity by decreasing the heterogeneity of soil phosphorus and carbon.

## 2 Results and Discussion

### 2.1 Spatiotemporal Scaling of Soil Bacterial Communities

**FIGURE 1:**
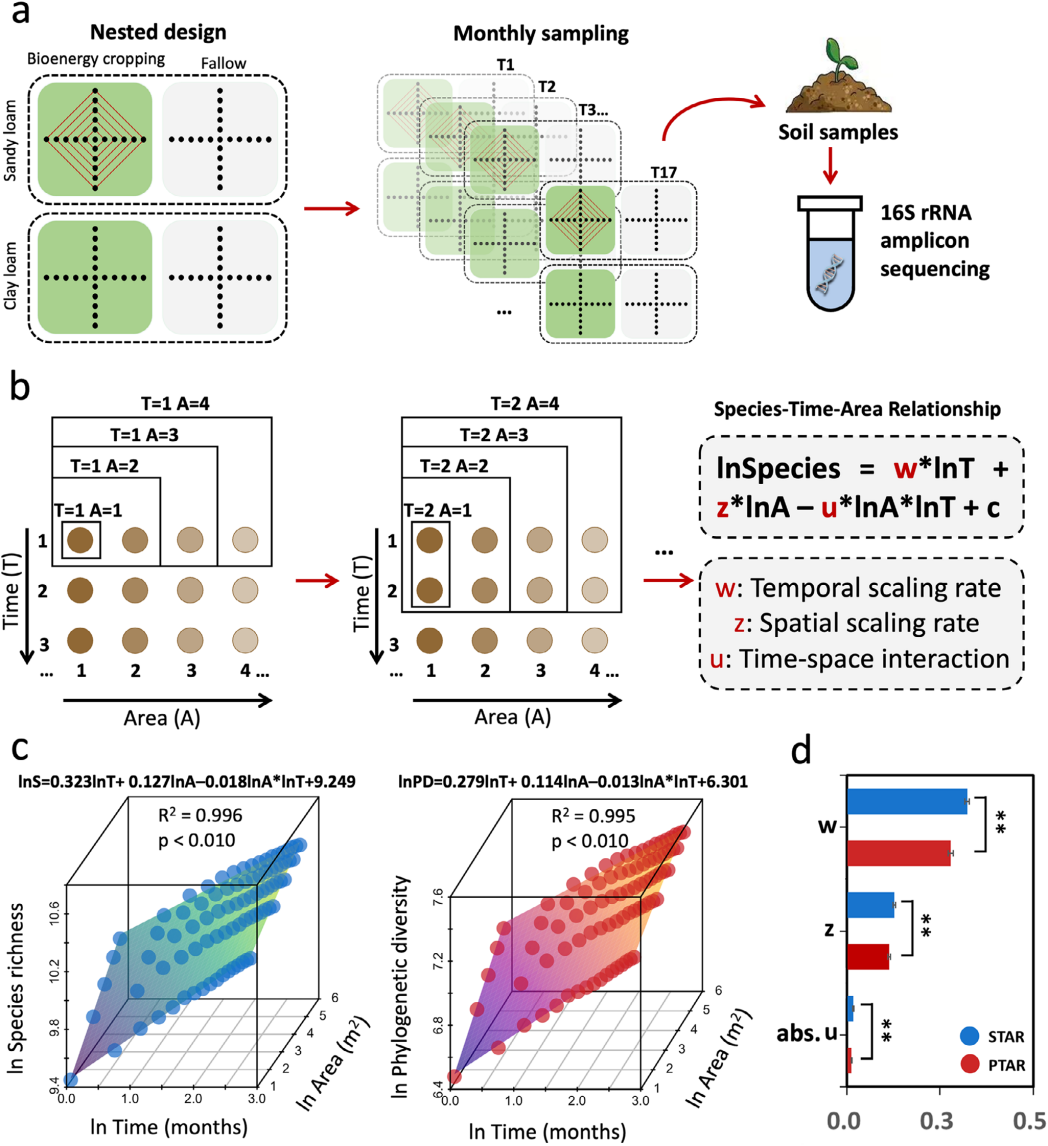
Bacterial diversity as a function of time, area, and a time–area interaction term. a, Experimental design. Two plots were established at each site: a switchgrass (bioenergy cropping) field plot and a corresponding fallow plot, resulting in four plots. At each plot, a nested design comprising 21 sampling points served as technical replicates used for nested scaling analyses. Monthly sampling from July 2016 to November 2017 was conducted for microbial sample collection. b, The nested method to calculate species‐time‐area relationship (STAR) and phylogenetic‐time‐area relationship (PTAR). In nested analyses, time is interpreted as scales of increasing size nested within each other, and the length of time is defined as the total time from the start of the initial survey. Specifically, for each time length (e.g., T0, T0+T1, T0+T1+T2 …), taxa in Area 1 was added to the number of new taxa in Area 2, then (1, 2) + new taxa in 3, (1, 2, 3) + 4, up to (1, 2, 3, 4) + 5, and then modeled richness (or phylogenetic diversity) of these taxa as a function of time length, area size, and time‐space interaction. c, The STAR and PTAR patterns in soil bacterial communities in bioenergy cropping plots at the sandy loam site. The parameters of both the STAR and PTAR models are significant (p < 0.010). The significance was examined by the permutation test (rand = 1,000). S: species richness; PD: phylogenetic diversity; A: area; T: time length. d, The difference in bacterial spatiotemporal scaling rates between taxonomic and phylogenetic diversity. The significance was examined by the permutation test (rand = 1,000). *** p < 0.001; ** p < 0.010; * p < 0.050. abs: the absolute value. The w: temporal scaling rate, z: spatial scaling rate, u: the time‐space interaction term.

We implemented nested sampling in bioenergy cropping and fallow plots and collected soil samples over a 17‐month period for 16S rRNA gene amplicon sequencing (Figure 1a). Based on the spatial and temporal sampling, we first calculated single‐variable models to estimate time‐space interactions during the scaling of bacterial communities at the taxonomic and phylogenetic levels. There were significant SARs (slopes = 0.092–0.101, averaged 0.097; R^2^ = 0.225–0.288, p < 0.001) and phylogenetic‐area relationships (PARs; slopes = 0.088–0.093, averaged: 0.091; R^2^ = 0.215–0.314, p < 0.001) for soil bacterial communities in both bioenergy cropping and fallow plots at the clay loam and sandy loam sites (Figure S1a,b, detailed in Text S1). Similarly, there were significant STRs (slopes = 0.255–0.332, averaged: 0.289; R^2^ = 0.689–0.742, p < 0.001) and phylogenetic‐time relationships (PTRs; slopes = 0.229–0.313, averaged 0.269; R^2^ = 0.664–0.751, p < 0.001; Figure S2a,b, detailed in Text S2). These results revealed strong spatiotemporal patterns in soil bacterial communities. Additionally, there were significant negative correlations between spatial scaling rates and time length (p < 0.001, Figure S1c,d), and between temporal scaling rates and area size (p < 0.001, Figure S2c,d), confirming significant time‐space interactions [1].

To further investigate whether the observed significant accumulation of bacterial diversity over temporal and spatial scales simply reflected sampling artifacts, we tested the Passive Sampling Hypothesis, which posits that species richness increases with sampling effort because larger habitats inherently contain more individuals or a broader pool of colonists [30]. Our results indicated that even after standardizing the total number of bacterial sequences for each spatial or temporal scale [11], soil bacterial richness and phylogenetic diversity continued to increase significantly with both time and space (Figure S3). Therefore, the Passive Sampling Hypothesis could be rejected. Instead, the observed increase in diversity appears to reflect genuine ecological dynamics, likely driven by environmental heterogeneity, dispersal processes, and sustained microbial adaptation to shifting conditions [11].

We further integrated spatial and temporal dimensions into a unified framework by generating STAR and PTAR models that accounted for their interactions. This joint perspective of STAR can provide a more comprehensive understanding of species diversity patterns than considering either scale alone [1, 6], because incorporating both space and time dimensions increases the proportion of explanatory power for variations in diversity. Specifically, we calculated the accumulation of bacterial richness (or Faith's phylogenetic diversity, the phylogenetic analog of taxon richness, which is the sum of the total phylogenetic branch lengths based on the phylogenetic tree constructed) with area size over each time length (the time length is defined as the total time from the start of the initial survey), and then modeled richness (or phylogenetic diversity) as a function of time length, area size, and time‐space interaction (Figure 1b). Our results indicated a strong fit of soil bacterial data to the STARs (R^2^ = 0.986–0.996, p < 0.001) and PTARs (R^2^ = 0.984–0.998, p < 0.001) in both bioenergy cropping and fallow plots across the two sites (Figure 1c; Figure S4a,b), which outperformed the single‐variable models (i.e., SARs, STRs, PARs, and PTRs; R^2^ = 0.215–0.751; Figures S1 and S2). Therefore, soil bacterial communities exhibited significant, strong STAR and PTAR patterns (R^2^> 0.980, p < 0.001), supporting our Hypothesis i. Even after rectifying sequence numbers at each spatial and temporal scale, soil bacterial communities still exhibited significant STAR and PTAR patterns (examined by permutation test, rand = 1,000; Figure S5). Furthermore, the STAR and PTAR exponents (w, z, and u values) remained significant in most cases (examined by permutation test, rand = 1,000; Figure S5), except for the PTAR exponents in the bioenergy‐cropping plots. However, the estimated scaling exponents and the time–space interaction term u changed in magnitude, suggesting that construction‐induced dependence may introduce uncertainty in exponent estimates, while having a comparatively smaller influence on the model significance. These findings indicate that the observed STAR and PTAR patterns are not solely caused by passive sampling effects. Therefore, by extending these relationships from plants and animals to soil bacterial communities, our findings suggest that STAR and PTAR can be regarded as universal ecological laws that extend beyond dependence on nested structure.

In the STAR and PTAR models, the area slopes (z‐values) represent spatial scaling rates per unit time, while the time slopes (w‐values) represent temporal scaling rates per unit area (Figure 1b). The PTAR z‐values (averaged 0.124) were smaller than the STAR z‐values (averaged 0.139) (p < 0.050), and the PTAR w‐values (averaged 0.331) were smaller than the STAR w‐values (averaged 0.366; p < 0.050; examined by permutation test, rand = 1,000; Figure 1d; Figure S4c), indicating that the taxonomic divergence of these communities was faster than phylogenetic divergence over space and time. This is due to the reason that the phylogenetic Faith's PD accumulates more slowly than species richness. Alternatively, it can be caused by the lower phylogenetic diversity of the regional species pool [16]. The slopes of SAR (or STR) were also higher than those of PAR (or PTR) (Figures S1 and S2).

Consistent with the observations found in animals and plants [6], the interaction terms in both the STAR and PTAR models (i.e., u‐values) were consistently negative (Figure 1c; Figure S4a,b), indicating that spatial scaling rates decreased with increasing time period (Figure S1c,d) and temporal scaling rates decreased with increasing area size (Figure S2c,d). Thus, the next question is what factors drive the decrease in spatial (or temporal) scaling as time (or area) increases? Given a static and finite regional species pool, it is expected that as sampled richness approaches the species pool's limit as the spatial or temporal scale increases, the rate of species accumulation slows due to the diminishing availability of rare species [31, 32, 33]. In other words, the percentage of newly discovered species decreases relative to the growing number of sampled species. Moreover, smaller spatial and temporal scales are more prone to stochastic events such as drift compared to larger scales [34, 35], which promotes dynamic changes in the bacterial communities and thus increases the scaling rates.

### 2.2 Changes in STAR and PTAR Across Different Bacterial Lineages

**FIGURE 2:**
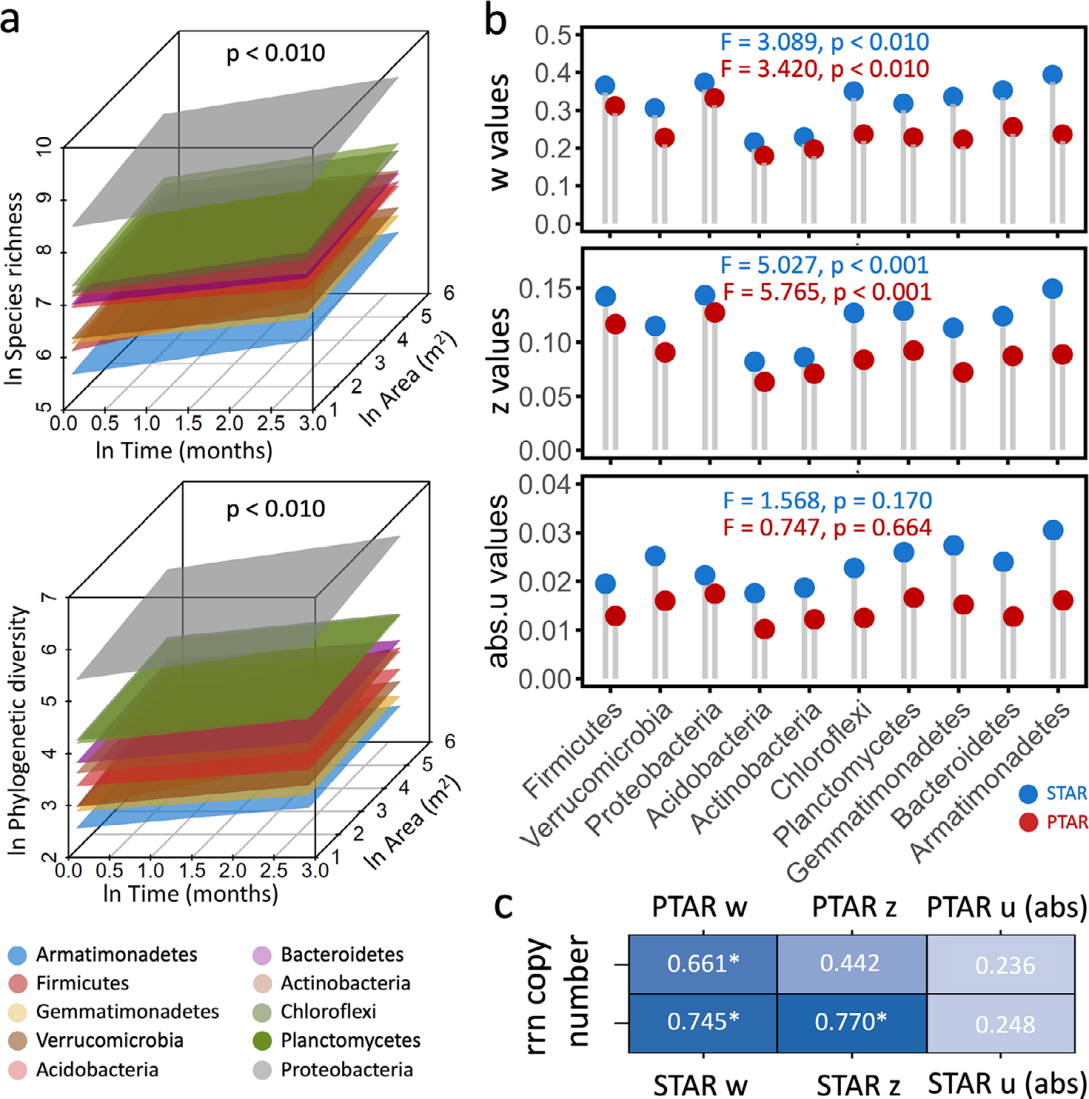
The spatiotemporal scaling patterns in different bacterial phyla. a, The species‐time‐area relationships (STARs) and phylogenetic‐time‐area relationships (PTARs) in different bacterial phyla in bioenergy cropping plots at the sandy loam site. The significance was examined by the permutation test (rand = 1,000). b, The difference in spatiotemporal scaling rates among different bacterial phyla, which was examined by ANOVA. Blue: STAR exponents; Red: PTAR exponents. c, The correlation between spatiotemporal scaling rates of different bacterial phyla and their rrn copy numbers (examined by the Spearman correlation method). The rrn copy numbers for bacterial operational taxonomic units (OTUs) were estimated based on the rrnDB database (version 5.4). For each bacterial phylum, the community‐level rrn copy number was calculated as the mean of the estimated rrn copy number, weighted by the relative abundance for each OTU. *** p < 0.001; ** p < 0.010; * p < 0.050. abs: the absolute value. The w: temporal scaling rate, z: spatial scaling rate, u: the time‐space interaction term.

We also observed significant STAR and PTAR patterns at the bacterial phylum level (p < 0.001; Figure 2a; Figure S6a) across all plots. There were substantial variations in the averaged spatiotemporal scaling rates based on taxonomic and phylogenetic diversity among different bacterial phyla (p < 0.010, examined by ANOVA; Figure 2b). For instance, *Acidobacteria* and *Actinobacteria* had considerably lower taxonomic and phylogenetic spatiotemporal scaling rates than *Firmicutes* and *Proteobacteria* (Figure 2b), which could be related to their different lifestyle strategies [9]. Thus, we further calculated the 16S ribosome RNA operon (*rrn*) copy numbers for these bacterial phyla (Figure S6b) because the *rrn* copy number is a reliable predictor of the growth rate and nutrient use efficiency of individual organisms [36]. There were significant positive correlations between spatiotemporal scaling rates (except for PTAR z) of bacterial phyla and their *rrn* copy numbers (Figure 2c). Taxa with high *rrn* copy numbers typically exhibit fast growth rates [36, 37]. Because a greater growth rate is often associated with higher reproductive rates and shorter generation times, accumulation of genetic variation over evolutionary time could be accelerated, producing faster replacement and consequently, higher spatiotemporal scaling rates. However, because rrn copy numbers were inferred by taxonomic matching to the rrnDB database and imputed to higher ranks when exact matches were unavailable, these estimates should be interpreted with caution as approximate trait proxies rather than precise values for each OTU. Matching uncertainty increases when taxa are resolved only to coarse ranks, which may affect the strength of the observed correlations. Similar to the patterns observed at the whole community level, the phylogenetic spatiotemporal scaling rates were lower than taxonomic spatiotemporal scaling rates across all bacterial phyla (Figure 2b; Table S1) due to the slower accumulation of phylogenetic diversity. Additionally, the changes in SAR (or PAR) and STR (or PTR) slopes among different bacterial phyla matched the overall trends observed in STAR (or PTAR) exponents (Table S2, detailed in Text S3, Supporting Information).

### 2.3 Effects of Bioenergy Cropping on Bacterial STARs and PTARs

**FIGURE 3:**
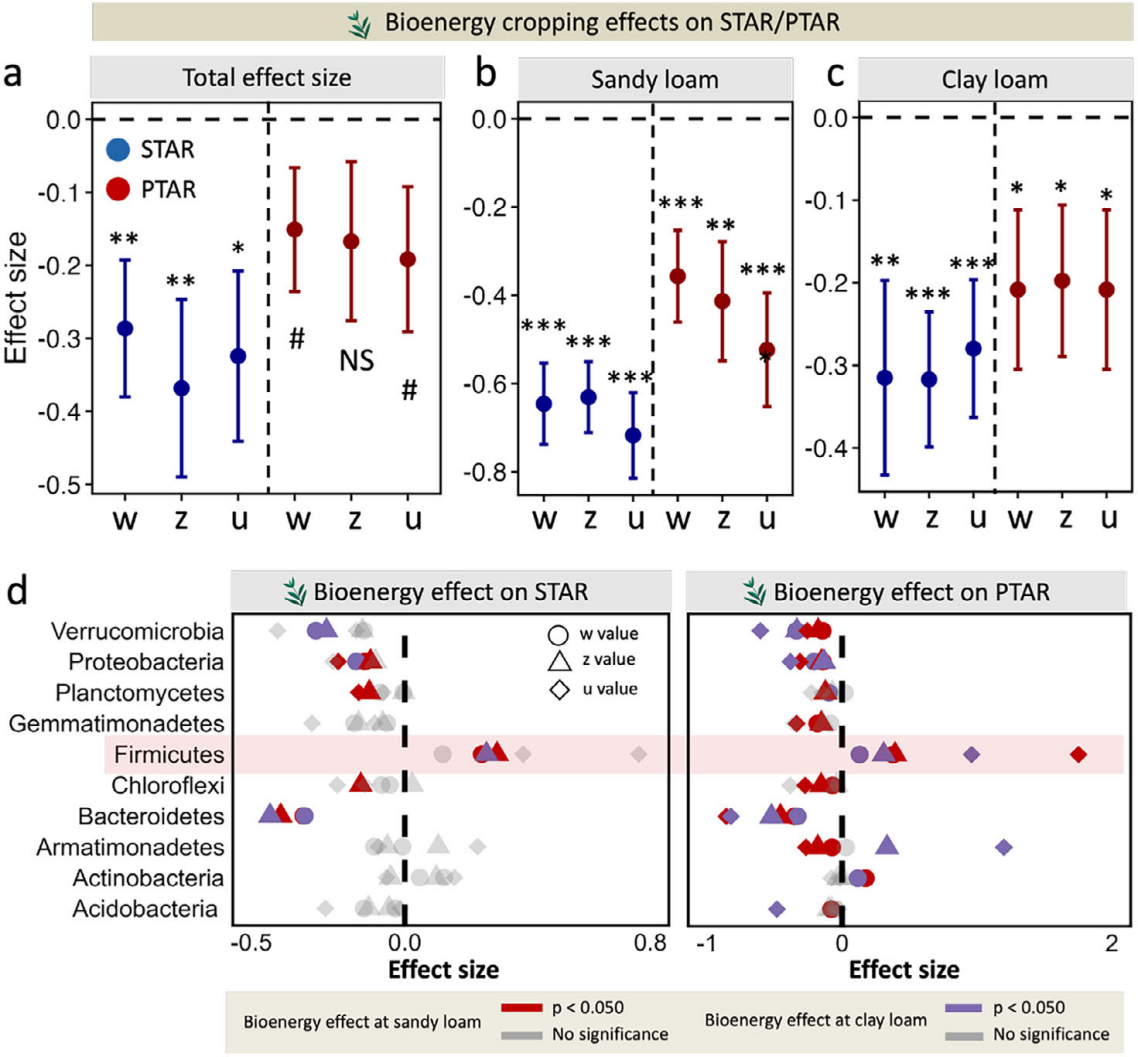
Effect of bioenergy cropping on the bacterial spatiotemporal scaling rates. a, The total effect size of bioenergy cropping on the bacterial taxonomic and phylogenetic spatiotemporal scaling rates (comparing bioenergy cropping with fallow lands; examined by linear mixed model, setting the site, plot ID, and time window size as random factors). b, The effect size of bioenergy cropping at the sandy loam site (examined by linear mixed model, setting the time window size as a random factor). c, The effect size of bioenergy cropping at the clay loam site (examined by linear mixed model, setting the time window size as a random factor). d, The effect of bioenergy cropping on taxonomic and phylogenetic spatiotemporal scaling rates among different bacterial phyla at clay loam and sandy loam soils. The effects are presented as the relative changes of (bioenergy cropping‐fallow)/fallow. The significance was examined by the permutation test (rand = 1,000), with gray representing insignificance (p ≥ 0.050). For panels a‐c, the error bar represents standard error. The w: temporal scaling rate, z: spatial scaling rate, u: the time‐space interaction term. Here, the u value is the absolute value. *** p < 0.001; ** p < 0.010; * p < 0.050; # p < 0.1; NS: no significance.

To assess how bioenergy cropping influences the spatiotemporal scaling rates of soil bacterial communities, we used a linear mixed model to estimate the effect size of bioenergy cropping. As expected, bioenergy cropping significantly reduced the taxonomic and phylogenetic spatiotemporal scaling rates (STAR w‐, STAR z‐, and PTAR w‐values) and time‐space interaction terms (the absolute values of STAR and PTAR u) when accounting for the two sites (Figure 3a), supporting our Hypothesis ii. When each site was considered individually and the time window size was included as a random factor, bioenergy cropping had significantly negative effects on the taxonomic temporal and spatial scaling rates and time‐space interaction terms at sandy loam site (β = ‐0.646 for STAR w, ‐0.631 for STAR z, and −0.717 for the absolute values of STAR u; p < 0.001, calculated by linear mixed model), and negative effects were also significant for the phylogenetic spatiotemporal scaling rates and time‐space interaction terms (β = −0.357 for STAR w, −0.413 for STAR z, and −0.523 for the absolute values of STAR u; p < 0.010, Figure 3b). A closer examination showed that bioenergy cropping significantly reduced the taxonomic and phylogenetic spatiotemporal scaling rates and time‐space interaction terms at the clay loam site (Figure 3c). Such negative effects of bioenergy cropping on bacterial scaling patterns were further corroborated by response ratio analyses (Table S3, Supporting Information). Interestingly, the effect size of bioenergy cropping on STAR and PTAR exponents at the sandy loam site was 15.0%‐161.0% larger than that at the clay loam site (Figure 3b,c), suggesting that the reduction in spatiotemporal scaling rates by bioenergy cropping was greater in sandy loam than in clay loam soils. It was likely that the higher soil nutrient and moisture levels in clay loam soil (Table S4, Supporting Information) partly offset the negative effects of bioenergy cropping on the spatiotemporal scaling rates of bacteria because greater resource supply can increase niche availability and promote coexistence [25], while the lower soil nutrient and moisture levels in sandy loam soil (Table S4, Supporting Information) exacerbated such negative effects of bioenergy cropping.

The bioenergy cropping effects on the spatiotemporal scaling rates varied considerably among different bacterial lineages. Bioenergy cropping had significant negative effects on the taxonomic and phylogenetic spatiotemporal scaling rates of most bacterial phyla, with the largest negative effects observed in *Bacteroidetes* (Figure 3d). However, bioenergy cropping had significant, positive effects on the spatiotemporal scaling rates of *Firmicutes* (Figure 3d), which had high *rrn* copy numbers (Figure S6b) and were usually considered as copiotrophic taxa thriving in nutrient‐rich environments [37, 38]. Thus, the increased soil nutrient availability due to bioenergy cropping (Table S4, Supporting Information) could promote the spatiotemporal scaling rates of *Firmicutes*. We also speculate that taxa affiliated with Firmicutes, capable of sporulation [39], persist in low abundances during unfavorable conditions and bloom under favorable conditions, thereby increasing scaling rates under bioenergy cropping.

**FIGURE 4:**
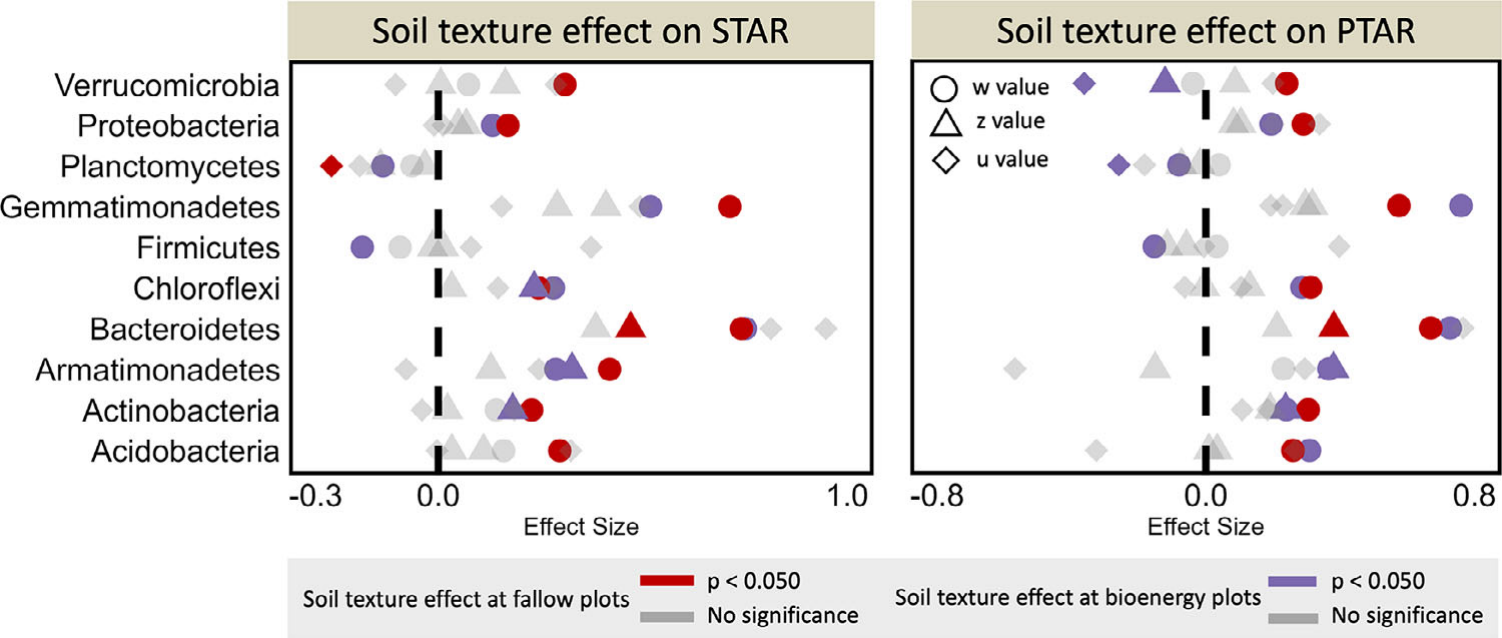
Effect of soil texture on the bacterial spatiotemporal scaling rates at the phylum level. The soil texture effects were calculated in the bioenergy cropping and fallow plots. The effects are presented as the relative changes of (clay loam – sandy loam)/sandy loam. The significance was examined by the permutation test (rand = 1,000), with gray representing insignificance (p ≥ 0.050). The w: temporal scaling rate, z: spatial scaling rate, u: the time‐space interaction term. Here, the u value is the absolute value.

We also calculated the effect size of soil texture (comparing clay loam soil to sandy loam soil) on bacterial spatiotemporal scaling rates by using a linear mixed model. The taxonomic and phylogenetic STAR and PTAR exponents in clay loam soil were significantly higher than those in sandy loam soil, whether the treatments (bioenergy cropping and fallow) were combined or separated (p < 0.050, linear mixed model; Table 1). Among these model exponents, the effects of soil texture on temporal scaling rates were the most pronounced, suggesting that bacterial temporal scaling may be more sensitive to soil conditions than spatial scaling. Interestingly, the effect size of soil texture was smaller in bioenergy cropping plots than in fallow plots (Table 1), suggesting that bioenergy cropping reduced the difference in bacterial spatiotemporal scaling between clay loam and sandy loam soils. This may be due to that the bacterial communities in bioenergy cropping plots were subjected to the influence of switchgrass root exudates [40], thereby reducing the differences in bacterial communities between sandy loam and clay loam soils. Similar to the patterns observed at the community level, the w‐values of most bacterial phyla were significantly higher at the clay loam site than sandy loam site (p < 0.050, examined by permutation test, rand = 1,000; Figure 4), while there was no significant difference in the z‐ and u‐values of most phyla between the two soil textures (Figure 4).

**TABLE 1:** The soil texture effect on bacterial spatiotemporal scaling. The effect size was calculated by the effect of clay loam soil on bacterial STAR and PTAR exponents, compared with that of sandy loam soil. The effect size and significance were estimated with a linear mixed model. When calculating the total effect size, the treatment (bioenergy cropping and fallow), plot ID, and time window size were considered as random factors. For calculating the effect size of soil texture in each treatment, the time window size was considered as a random factor. A positive effect indicates a larger STAR or PTAR exponents compared to the control treatment, with the reciprocal being true for negative effects. The w: temporal scaling rate, z: spatial scaling rate, u: the time‐space interaction term.

		Total effect size		Bioenergy plots		Fallow plots	
	Exponents	Effect size	p value	Effect size	p value	Effect size	p value
STAR	w	1.090	<0.001	0.962	<0.001	1.270	<0.001
	z	0.630	<0.001	0.564	<0.001	0.754	<0.001
	u	0.304	<0.050	0.271	<0.050	0.353	<0.010
PTAR	w	1.150	<0.001	1.040	<0.001	1.358	<0.001
	z	0.475	<0.001	0.330	<0.010	0.712	<0.001
	u	0.487	<0.001	0.372	<0.010	0.634	<0.001

Similar patterns were observed for the SAR (or PAR) and STR (or PTR) slopes (Figure S7). For instance, bioenergy cropping reduced the SAR (or PAR) and STR (or PTR) slopes of bacterial communities and their lineages (Figure S7a), with these slopes being higher at the clay loam site than at the sandy loam site (Figure S7b and Text S4). Collectively, these results indicated that bioenergy cropping could alleviate the rapid changes in soil biodiversity over space and time, thereby contributing to the stability of bacterial communities [41]. To validate this, we quantified the temporal stability of bacterial richness as an index of stability. Temporal stability was significantly negatively correlated with the temporal scaling rates based on taxonomic diversity (Figure S8), indicating that higher temporal scaling was associated with reduced bacterial community stability.

We reanalyzed the data using the DADA2 pipeline to evaluate the sensitivity of the STAR exponents and treatment contrasts to different 16S processing approaches. Consistent with the results from 97% OTU clustering (Figure 1c; Figure S4a), the DADA2 pipeline confirmed that the STAR exponents were significant and exhibited negative responses to bioenergy cropping (Figure S9b), which indicates that our conclusions are robust to the choice of microbial analysis pipeline. But the values of exponents derived from the DADA2 pipeline (Figure S9a) differed to some extent from those based on 97% OTU clustering (Figure 1c; Figure S4a), reflecting pipeline‐dependent richness biases.

### 2.4 Mechanisms Underlying Changed Bacterial Spatiotemporal Scaling Rates

**FIGURE 5:**
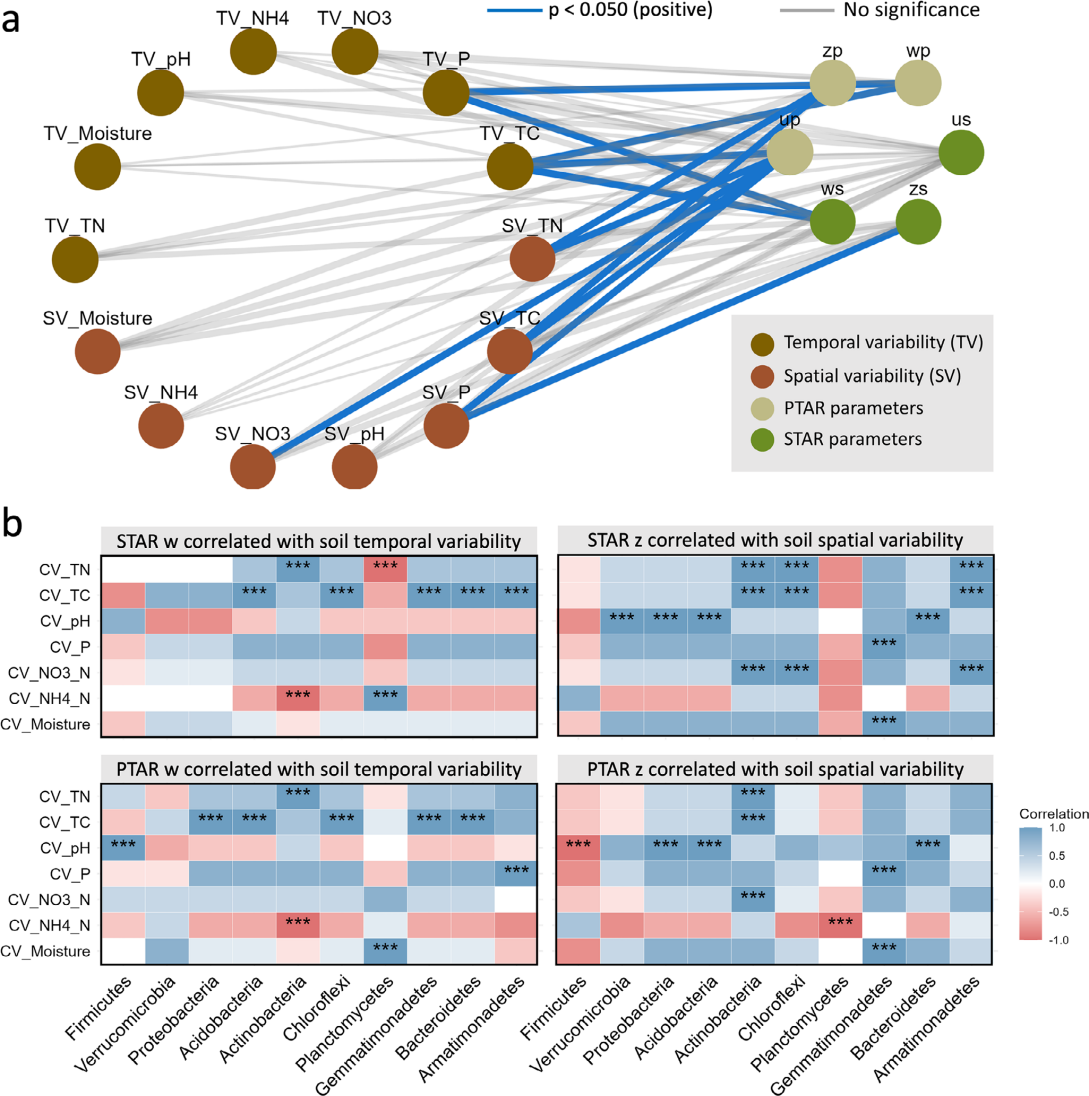
Correlation between bacterial spatiotemporal scaling rates and soil properties. a, The correlation between the variability of soil properties and spatiotemporal scaling rates of bacterial community. The variability (coefficient of variation; CV) was treated as the soil heterogeneity. The STAR and PTAR exponents were fitted to linear regression models with the variability of soil property as an independent variable. The temporal scaling rates were fitted with the temporal variability of soil properties, while the spatial scaling rates were fitted with the spatial variability of soil properties. The absolute u values were fitted with the spatial and temporal variabilities of soil properties. The blue line represents a positive correlation with p < 0.050, and the gray line represents p ≥ 0.050. b, The correlation between the variability of soil properties and spatiotemporal scaling rates of different bacterial phyla, which was examined by the Spearman correlation method. The p‐values were adjusted by the FDR method. *** p < 0.001; ** p < 0.010; * p < 0.050. The ws: taxonomic‐based temporal scaling rate, zs: taxonomic‐based spatial scaling rate, us: the taxonomic‐based time‐space interaction term. The wp: phylogenetic‐based temporal scaling rate, zp: phylogenetic ‐based spatial scaling rate, up: the phylogenetic‐based time‐space interaction term.

Bioenergy cropping‐induced decrease in bacterial spatiotemporal scaling rates contradicts the species–energy theory, which posits that the energy available to a community constrains its species [24], indicating that the spatiotemporal scaling of soil bacterial communities is not dependent on the increased resources and energy inputs derived from bioenergy cropping (Table S4, Supporting Information). Such reductions may be due to the changes in soil heterogeneity [11]. Under bioenergy cropping conditions, spatial and temporal heterogeneity (i.e., coefficient of variation) of most soil properties were reduced (Figure S10; Cohen's d < ‐0.200), which might be due to the reduced above‐ground plant heterogeneity following single bioenergy cropping. Consequently, the number of available niches would be substantially decreased [42, 43], which could lead to a reduction in the number of coexisting species according to the Environmental Heterogeneity Hypothesis [11] positing that greater habitat heterogeneity provides more ecological niches, enabling a greater range of species to coexist at a given spatial or temporal scale [11]. Eventually, the bioenergy cropping‐induced reduction in species coexistence could reduce the bacterial spatiotemporal scaling rates. These inferences were supported by the significant, positive correlation between taxonomic and phylogenetic bacterial spatiotemporal scaling rates (w‐ and z‐values) and heterogeneity of soil P and TC (CV, Figure 5a), a pattern that was further supported by results based on Euclidean distance metrics of soil heterogeneity (Figure S11). Similarly, the changes in SAR (or PAR) and STR (or PTR) slopes were also correlated with the soil heterogeneity (Figure S12, detailed in Text S5). However, this study cannot rule out potential influences from other unmeasured factors, such as available potassium, plant available phosphorus, and trace elements. In addition, other potential drivers may also contribute to the observed patterns. For instance, bioenergy cropping could act as a deterministic filtering factor to impose strong selection on soil microorganisms due to the exudation of root exudates [44], which would lead to a reduction in the size and heterogeneity of the species pool and even more specialized microbial taxa such as nitrogen‐fixing [40] and phosphate‐solubilizing [45] bacteria, further reducing the bacterial spatiotemporal scaling rates. Nutrient enrichment following the establishment of bioenergy crops is also likely to shift soil bacterial communities toward more copiotrophic assemblages, particularly during the later stages of cultivation (Figure S13). These taxa can rapidly capitalize on increased resource availability, quickly occupying available niches and thereby reducing biodiversity scaling. In addition, bioenergy cropping increased bacterial immigration rates (Figure S14; examined by linear mixed model, p < 0.050), which could homogenize bacterial communities across space and time and consequently reduce scaling rates. According to the “Hunger Games” hypothesis [37], improved nutrient levels by bioenergy cropping (Table S4) may intensify competitive exclusion among microbes, weakening coexistence and thus leading to lower scaling rates. However, the current experimental design does not allow us to disentangle which is dominant or how they might interact.

According to the Environmental Heterogeneity Hypothesis [11], the higher soil heterogeneity in clay loam than in sandy loam soils (Figure S10) could lead to higher bacterial spatiotemporal scaling rates in clay loam soils (Table 1; Figure 4). Moreover, clay loam soils contain a higher proportion of fine clay particles than sandy loam soils, yielding a larger specific surface area that creates a greater diversity of microhabitats [46]. Clay loam soils also typically contain more micropores with weaker pore connectivity than sandy loam soils, increasing spatial isolation of bacteria within microstructures and strengthening dispersal limitation [47]. These properties of clay loam soils could accelerate the spatiotemporal scaling of bacterial diversity. For the absolute values of u, the taxonomic‐based u values showed no significant correlations with soil heterogeneity, whereas the phylogenetic‐based u values were significantly positively correlated with both the temporal and spatial heterogeneity of soil TC (Figure 5a).

We also observed significantly positive correlations between the spatiotemporal scaling rates of most bacterial phyla and soil variables, but the driving factors varied among bacterial phyla (Figure 5b). For instance, the STAR and PTAR w‐values of *Acidobacteria*, *Bacteroidetes*, *Chloroflexi*, and *Gemmatimonadetes* were positively correlated with the temporal heterogeneity of TC, while those of *Actinobacteria* were positively correlated with the temporal heterogeneity of TN (p < 0.001; Figure 5b). The STAR and PTAR z‐values of *Proteobacteria*, *Acidobacteria*, and *Bacteroidetes* were positively correlated with the spatial heterogeneity of pH, while those of *Gemmatimonadetes* were positively correlated with the spatial heterogeneity of P (p < 0.001; Figure 5b). These results indicate that different bacterial phyla exhibit distinct preferences for environmental niches. Also, the time‐space interaction terms (the absolute values of u) in half of these bacterial phyla were positively affected by pH heterogeneity (Figure S15), a primary factor in shaping soil microbial communities [48]. Given that soil heterogeneity has been widely reported as an important determinant of microbial community composition across ecosystems [42, 49], our finding that bioenergy cropping decreased bacterial spatiotemporal scaling through reduced soil heterogeneity suggests that this response should be most transferable to comparable bioenergy cropping systems or agricultural ecosystems that similarly promote soil homogenization [50].

An intriguing question is whether the bioenergy cropping‐reduced spatiotemporal scaling rates affect the ecosystem processes. We focused on soil methane (CH_4_) flux because it is a potent greenhouse gas, and both its production and oxidation are tightly coupled to soil microbial communities and organic matter decomposition [51]. Our results showed that the temporal scaling rate of soil bacterial taxonomic diversity was significantly and negatively correlated with CH_4_ flux, whereas the spatial scaling rate showed no significant correlation (Figure S16). Given that these soils act as a net methane sink in our study [22], this pattern suggests that the reduced temporal scaling rate of bacterial diversity by switchgrass cropping may be accompanied by weaker CH_4_ uptake, potentially even shifting soils from a net CH_4_ sink to a net CH_4_ source (Figure S16).

### 2.5 Comparison of Bacterial STARs/PTARs to Other Organisms

To gain broader insights into the spatiotemporal scaling of biodiversity across different organisms, we compared bacterial spatiotemporal scaling rates from this study with published data from other organisms (Figure S17a–c). Given the differences in sampling and analytical methods [9], direct comparison across studies is challenging [7, 10]. Therefore, we only made coarse‐level comparisons. Consistent with previous findings in STRs [9], we found higher bacterial STAR w‐values (i.e., temporal scaling rate per unit area) compared to those of plants (grass) and algae (Figure S17a). However, the STAR w‐values of small mammals and birds were considerably higher than those of soil bacteria (Figure S17a). A potential explanation is that the strictly timed migration of birds [52] and hibernation of small mammals [53] contribute to high temporal scaling in these organisms. In addition, we observed lower bacterial STAR z‐values (i.e., spatial scaling rate per unit time) compared to those of most other organisms (Figure S17b), consistent with the findings previously reported in SARs [4, 54, 55]. This is reasonable since bacteria are characterized by small body size, massive population sizes, rapid asexual reproduction, and high resistance to extinction [56], all of which facilitate their dispersal and migration, resulting in low STAR z_‐_values. The positive correlations between SAR slopes and body size in living organisms also support this inference [57, 58]. Similarly, the absolute STAR u‐values (i.e., time‐space interaction terms) of soil bacteria were lower than those of most organisms (Figure S17c), suggesting that soil bacterial diversity was less influenced by time‐space interactions compared to other organisms. However, very few studies have examined PTARs in plants, animals, and microbial organisms [59].

### 2.6 Time‐Space Equivalence

The mutual dependence of spatial scaling (or temporal scaling) and time period (or area size) (Figures S1c,d and S2c,d) suggests that the z‐ and w‐values can be equivalent [1], which illuminates the scales at which spatial and temporal processes converge. Therefore, we estimated the time‐space equivalence ratio based on the exponents of STAR models for bacterial communities at our study sites (Figure S18a). The richness‐based equivalence ratios ranged from 0.216 to 1.020 km^2^/yr (Figure S18a and Table S5), indicating that the bacterial species accumulated over one year was equivalent to that detected when sampling an area with a size of 0.216 – 1.020 km^2^ was sampled in the present study. Therefore, for a given area, species diversity in a future time point can be roughly estimated by extrapolating to larger spatial scales, effectively enabling a space‐for‐time substitution [9]. Such extrapolation provides a useful first‐order estimate of future diversity; however, the estimated spatiotemporal equivalency ratio requires further validation with empirical sampling, and more accurate predictions would require incorporating additional factors such as environmental heterogeneity.

The equivalence ratios were higher in bioenergy cropping plots (0.648 km^2^/yr for the sandy loam site and 1.020 km^2^/yr for the clay loam site) than in fallow plots (0.216 km^2^/yr for the sandy loam site and 0.926 km^2^/yr for the clay loam site) when controlling for consistent soil textures (Table S5, Supporting Information). Since a larger ratio indicates a stronger dispersal ability [6], these results suggested higher bacterial dispersal in bioenergy cropping plots than in fallow plots [60]. This finding was further validated by the neutral community model, which revealed that bioenergy cropping enhanced the immigration rates of soil bacterial communities (Figure S14).

To explore time‐space equivalence across organisms, we made coarse‐level comparisons of the soil bacterial equivalence ratio from this study with published data (Figure S18b; converting to the same unit m^2^/yr). The averaged equivalence ratio of soil bacteria calculated in this study is 7.014 × 10^5^ m^2^/yr (based on richness), which is much higher than the averaged ratios of plants (grass; 1.798 × 10^3^ m^2^/yr), invertebrates (4.100 m^2^/yr), rodents (8.000 × 10^3^ m^2^/yr) and mammals (small and large mammals; 1.160 × 10^5^ m^2^/yr), but lower than the averaged ratios of birds (1.445 × 10^11^ m^2^/yr) and zooplankton (4.600× 10^9^ m^2^/yr) (Figure S18b). These results suggest that soil bacteria may be more mobile and widely dispersed than most other organisms. However, birds and zooplankton, which move quite easily in their environments, exhibit higher equivalence ratio values than soil bacteria (Figure S18b).

### 2.7 Limitations

A major limitation of our study is the relatively short temporal scale considered, which constrains our ability to assess the longer‐term effects of bioenergy cropping and to infer how these responses may evolve under sustained climatic change. Long‐term observations will therefore be essential to determine whether the patterns reported here persist, strengthen, or reverse through interannual variability. Another limitation of this study is that it focuses on a single bioenergy cropping system and limited marginal soil types in a small study area. Future work should evaluate other bioenergy crops and management strategies on different marginal soils, including monocultures and mixed‐species plantings, to test whether our conclusions generalize across bioenergy systems. Finally, the limited number of sites and plots in this study constrains the strength of inference regarding bioenergy cropping effects. Expanding site and plot replication in future work would enhance the robustness and generality of these findings.

## 3 Conclusion

Understanding the spatial and temporal scaling of biological communities, along with the underlying mechanisms, is a fundamental issue in ecology. However, no research has yet explored the relationships between species richness and time‐space interactions in microbial communities in nature. Our study provides an examination of the spatiotemporal scaling of soil bacterial communities, revealing that STARs and PTARs exist for bacteria and their lineages independently of sampling effort within the studied system, and suggesting that STARs and PTARs could be universal laws in ecology [1, 6, 61]. Additionally, our results showed that bioenergy cropping significantly reduced both the taxonomic and phylogenetic spatiotemporal scaling rates of soil bacterial communities. This study represents the first empirical evidence of how bioenergy cropping affects the soil microbial biodiversity scaling across space and time. We also identified the time‐space equivalent scenario based on the exponents of STAR models, offering a new perspective for quantifying the scales of time‐space equivalence.

Our findings have important implications for biodiversity conservation and ecosystem management. First, microbial biodiversity depends on both the spatial and temporal scales at which it is assessed [6, 10], suggesting that determining the appropriate spatial and temporal scales for biodiversity assessment is important for ecosystem management [1]. Specifically: (i) conservation efforts should be tailored to the specific spatiotemporal dynamics of different lineages or evolutionary history [9, 62]; and (ii) the size of the priority conservation area and restoration timelines can be predicted based on the spatiotemporal scaling rates of microbial lineages [9, 16]. Since time‐space equivalent scenarios can be identified using STAR and PTAR models, space‐for‐time substitutions could be a valid and efficient approach for predicting long‐term biodiversity dynamics [63]. The space‐time equivalence ratio can also inform how conservation resources are allocated by indicating, for a given diversity restoration target, whether investments are more efficiently directed toward expanding protected areas or extending the duration of protection. Second, as demonstrated in this study, bioenergy cropping reduced bacterial spatiotemporal scaling rates regardless of soil textures. Such reductions could alleviate rapid changes in soil biodiversity and further enhance community stability [41], which could improve ecosystem functions, such as nutrient cycling and carbon sequestration [64], and make these communities more resilient to environmental changes. In addition, since temperature directly accelerates metabolic rates and biochemical processes [65, 66], biodiversity could change more quickly due to accelerated dynamic responses under future climate change scenarios [9, 55, 67], which could result in potential biodiversity loss [68]. Therefore, by demonstrating how bioenergy cropping interacts with soil microbial communities, this study provides evidence that such bioenergy systems could help alleviate biodiversity dynamics and maintain ecosystem functions, supporting their role as part of sustainable land management and climate mitigation strategies. However, such homogenization following bioenergy cropping, while reducing diversity scaling, may also lead to potential losses of diversity. Approaches such as appropriate mixed sowing or intercropping could help maintain higher diversity while still reducing diversity scaling.

## 4 Materials and Methods

### 4.1 Field Site and Sample Collection

Samples were collected from two locations in southern Oklahoma: a sandy loam site near the Texas border (33.881715°N, −97.275167°W) and a clay loam site in Ardmore (34.172100°N, −97.07953°W) [22, 69]. These sites were chosen due to their depleted soil nutrient levels and history of topsoil erosion, making them marginal lands. In the summer of 2016, two plots were established at each site: a bioenergy cropping field plot (27 × 22 m) containing 500 genetically distinct individuals of switchgrass (the lowland Alamo variety) spaced 1 m apart, and a paired fallow plot (27 × 22 m) (Figure 1a). Prior to the experiment, all plots were tilled to a depth of 30 cm. Then switchgrass seedlings were planted at 1 m intervals. The bioenergy cropping plots were sustainably managed without the use of chemical fertilizers, herbicides, or watering. Fallow plots were left to undergo a natural succession of grasses and weeds throughout the experiment, serving as controls. As a result, the fallow surface on the sandy loam site was mostly bare soil (∼52.0%), with plant litter (∼31.0%) and annual forbs (∼17.0%) covering the remaining area. The surface on the clay loam site was primarily occupied by annual grass species (∼86.0%), with annual forbs (∼2.0%) and bare soil (∼1.0%) covering the remaining area.

At each plot, a cross‐design (nested) comprising 21 sampling points (Figure 1a) served as technical replicates used for nested scaling analyses. Two soil cores (0–20 cm depth) were extracted from within a 20 cm radius of each sampling point, mixed thoroughly, and divided into two aliquots: one for geochemical analyses and one for DNA extraction. Sampling flags were deployed to prevent duplicate sampling, and each core was refilled with topsoil from outside the plot. Monthly sampling from July 2016 to November 2017 (17 months, covering 2 growing seasons) was conducted, yielding a total of 1,428 samples. All soil samples were promptly preserved on dry ice, transported to the laboratory within 5 h, and stored at either 5°C for geochemical analyses or −80°C for DNA extraction.

### 4.2 Soil Physiochemistry Measurments

Soil pH, moisture, total soil carbon (TC) and nitrogen (TN), plant‐available phosphorus (P), nitrate (NO_3_^−^), and ammonium (NH_4_^+^) pools were measured as previously described [22]. These soil properties were measured monthly during the first year (5 months, July 2016 to November 2016) and seasonally during the second year (4 months, which were March, June, September, and November 2017). The CH_4_ flux was measured monthly using cavity ring‐down spectrometry with a Picarro G2508 analyzer (Picarro, Santa Clara, CA, USA). For each collar, gas concentrations (ppm) were recorded every 2 s over a 6 min sampling period.

### 4.3 Microbiome Analyses

DNA extraction from a total of 1,428 soil samples was performed using a freeze grinding method in conjunction with the Powersoil DNA extraction kit (Qiagen Inc., Venlo, Netherlands), resulting in DNA of high quantity and quality. Microbial community profiling involved a two‐step PCR approach for amplifying the V4 region of the bacterial 16S rRNA gene, utilizing the 515F (5’‐GTGCCAGCMGCCGCGGTAA‐3’) and 806R (5’‐GGACTACHVGGGTWTCTAAT‐3’) primers. Sequence analyses were performed using the pipeline of Quantitative Insights Into Microbial Ecology (QIIME) pipeline (version 1.9.1), with dereplication carried out by USEARCH (version 9.2.64). Operational Taxonomic Units (OTUs) were clustered using UPARSE, applying a 97.0% similarity threshold after removing singletons/chimeric sequences. An OTU table was then generated using the USEARCH command. The raw paired‐end reads were also de‐noised, assembled using DADA2 pipeline, clustered into different amplicon sequence variants (ASVs), and then were subject to taxonomic assignment using the Ribosomal Database Project classifiers within the SILVA, and an ASV table was then generated.

### 4.4 Methods for Considering Time Length

There are three different methods to construct STRs by their definition of sample time; termed here as the “nested,” “complete nested,” and “island” [5, 70, 71]. In all of these types, species richness is plotted as a function of increasing length of sample time.

In nested analyses, time is treated similarly to species‐area studies, where areas of increasing size are nested within each other (Figure S19a). The length of the sample time is defined as the total time from the start of the initial survey. For example, in a 17‐timepoint time series, taxa richness in timepoint 1 is added to the number of new taxa in timepoint 2 (T = 2), then (1, 2) + new taxa in 3 (T = 3), (1, 2, 3) + 4 (T = 4), etc., up to (1, 2, 3 …. 15, 16) + new taxa in timepoint 17 (T = 17). Each data point represents a single value: total species richness in the sample of length of time calculated from the start of the initial survey.

The complete‐nested design, in contrast, defines the length of sample time as the average of every possible combination of consecutive sample time lengths (Figure S19b). For example, in a 17‐timepoint time series, the analysis is broken down into 17‐timepoint windows, 16 two‐timepoint windows, 15 three‐timepoint windows, etc. For the two time point windows (T = 2), taxa richness in time point 1 was added to the number of new taxa present in time point 2, the window was then moved onto time points 2 and 3, 3 and 4, etc., progressing until time points 16 and 17. For the three timepoint windows (T = 3), the taxa richness in time points 1 and 2 has the new taxa in timepoint 3 added, then (2, 3) + 4, (3, 4) + 5, etc., up to (15, 16) + 17. Window size continued to increase sequentially in that manner until arriving at the final seventeen point window, e.g., (1, 2, 3, … 15, 16) + new taxa in timepoint 17 (T = 17). Each data point represents the mean number of species across all samples of consecutive periods of length T, with time defined only as an interval.

STRs can also be constructed from temporal islands (Figure S19c), analogous to spatial islands [70]. Here, each data point represents a unique survey (temporal island) conducted for a length of sample time. The STR plots the total number of species observed in each survey as a function of the total length of that survey, encompassing multiple surveys of different lengths of time for the same area. Here, time is defined as an interval from isolated biological or sampling “islands.”

To match the nested spatial sampling, we calculated the STARs and PTARs by a nested method, which provides the best fit for species richness and time scale compared to the other methods according to a previous study [5]. Additionally, given that the complete‐nested design is another widely used approach for temporal scaling at ecological scales [9, 70] and accounts for seasonal variations, we also calculated STARs and PTARs by using the complete‐nested method and compared the differences in STAR (or PTAR) exponents between nested and complete‐nested methods (Figure S20). There were no significant differences in STAR (or PTAR) exponents between these two methods (p > 0.050, examined by t‐test; Figure S20), indicating that different considerations of time length in this study did not affect the main findings. Therefore, we selected nested‐based results for subsequent analyses to avoid redundant analysis.

### 4.5 Calculation of SARs, PARs, STRs, and PTRs

The SARs of bacteria are usually evaluated using the linear regression between logarithmic richness and logarithmic area in the following form Equation (1).

(1) $$\ln\left(S\right)=z_{s1}\ln\left(A\right)+b$$

where S is the richness, A is the area, the slope z_s1_ is the SAR value, a measure of the spatial scaling rate of the species richness, and b is the intercept. For each time length (e.g., T_0_, T_0_+T_1_, T_0_+T_1_+T_2_ …), taxa richness in Area 1 was added to the number of new taxa in Area 2, then (1, 2) + new taxa in 3, (1, 2, 3) + 4, up to (1, 2, 3, 4) + 5, considering the spatiotemporal interactions.

PARs, the phylogenetic analogs of SARs, were estimated using a similar logarithmic equation Equation (2):

(2) $$\ln\left(\mathrm{PD}\right)=z_{p1}\ln\;\left(A\right)+b$$

where PD is the phylogenetic diversity, A is the area, the slope z_p1_ is the PAR value, a measure of the spatial scaling rate of the phylogenetic diversity, and b is the intercept. For each time length (e.g., T_0_, T_0_+T_1_, T_0_+T_1_+T_2_ …), taxa richness in Area 1 was added to new taxa in Area 2, then (1, 2) + new taxa in 3, (1, 2, 3) + 4, up to (1, 2, 3, 4) + 5. For each time length, the Faith's phylogenetic diversity in each area was calculated based on packages “picante” and “ape” in R v4.2.1.

The STRs of bacteria are usually evaluated using the linear regression between logarithmic richness and logarithmic time in the following form Equation (3).

(3) $$\ln\left(S\right)=w_{s1}\ln\left(T\right)+b$$

where S is the richness, T is the length of time, the slope w_s1_ is the STR value, a measure of the temporal scaling rate of the species richness, and b is the intercept. In this study, the nested approach was used [5]. Specifically, for each area, taxa richness in timepoint 1 was added to the number of new taxa in timepoint 2, then (1, 2) + new taxa in 3, (1, 2, 3) + 4, (1, 2, 3, 4) + 5, etc., up to (1, 2, 3 …. 15, 16) + new taxa in 17, considering the spatiotemporal interactions.

PTRs, the phylogenetic analogs of STRs, were estimated using a similar logarithmic equation Equation (4):

(4) $$\ln\left(\mathrm{PD}\right)=w_{p1}\ln\left(T\right)+b$$

where S is the richness, T is the length of time, the slope w_p1_ is the PTR value, a measure of the temporal scaling rate of the phylogenetic diversity, and b is the intercept. The nested approach was used to calculate the PTR for each area. Specifically, for each area, taxa in timepoint 1 were added to new taxa in timepoint 2, then (1, 2) + new taxa in 3, (1, 2, 3) + 4, (1, 2, 3, 4) + 5, etc., up to (1, 2, 3 …. 15, 16) + new taxa in 17. For each area, the Faith's phylogenetic diversity over each time length was calculated based on the packages “picante” and “ape”.

We also calculated SARs, PARs, STRs, and PTRs without considering spatiotemporal interactions for common phyla observed within each plot (i.e., *Acidobacteria*, *Actinobacteria*, *Armatimonadetes*, *Bacteroidetes*, *Chloroflexi*, *Firmicutes*, *Gemmatimonadetes*, *Planctomycetes*, *Proteobacteria*, and *Verrucomicrobia*). To test the Passive Sampling Hypothesis, we standardized the total number of bacterial sequences for each spatial or temporal scale, and then fitted the SAR and STR models [11].

### 4.6 Calculation of STAR and PTAR

A generalized power‐law model was proposed to effectively capture the covariation of species richness with space and time [6] (the species‐time‐area relationship; STAR; Equation (5):

(5) $$\ln\left(S\right)=z_{s}\ln\left(A\right)+w_{s}\ln\left(T\right)+u_{s}\ln\left(A\right)\ln\left(T\right)+b$$

Here, S is the richness, A is the area, T is the time length, z_s_ represents the slope of the SAR over one month, w_s_ denotes the slope of the STR at a spatial scale of 1 m^2^, u_s_ quantifies the interaction between area and time in determining richness, and b stands as a constant. In order to maintain consistency with STR calculations, the nested method was also used in this study to calculate STAR. For each time length (e.g., T_0_, T_0_+T_1_, T_0_+T_1_+T_2_ …), taxa richness in Area 1 was added to the number of new taxa in Area 2, then (1, 2) + new taxa in 3, (1, 2, 3) + 4, up to (1, 2, 3, 4) + 5. We then fitted the richness with time length and area, considering the time‐space interaction.

We further expand STAR to PTAR, that is, the phylogenetic‐time‐area relationship (equation (6)).

(6) $$\ln\left(\mathrm{PD}\right)=z_{p}\ln\left(A\right)+w_{p}\ln\left(T\right)+u_{p}\ln\left(A\right)\ln\left(T\right)+b$$

Here, PD is the phylogenetic diversity, A is the area, T is the time length, z_p_ is the slope of the PAR at the temporal scale of one month, w_p_ is the slope of the PTR given a spatial scale of 1 m^2^, u_p_ measures the interaction between area and time in determining phylogenetic diversity, and b is a constant. For each time length (e.g., T_0_, T_0_+T_1_, T_0_+T_1_+T_2_ …), taxa richness in Area 1 was added to the new taxa in Area 2, then (1, 2) + new taxa in 3, (1, 2, 3) + 4, up to (1, 2, 3, 4) + 5. For each time length, the Faith's phylogenetic diversity in each area was calculated based on the packages “picante” and “ape”. We then fitted the phylogenetic diversity with time length and area, considering the time‐space interaction. We also calculated STARs and PTARs for common phyla observed within each plot (i.e., *Acidobacteria*, *Actinobacteria*, *Armatimonadetes*, *Bacteroidetes*, *Chloroflexi*, *Firmicutes*, *Gemmatimonadetes*, *Planctomycetes*, *Proteobacteria*, and *Verrucomicrobia*).

Based on the STAR and PTAR models, we calculated the scales of time–area equivalence [18]. Scales of equivalence are defined as the combination of sampled area and time period, where the slopes of the STR/PTR and SAR/PAR (i.e., scaling rates) are equivalent. Scales of equivalence are usually presented as ratios, and using the estimated exponents from the interaction STAR/PTAR model, they are calculated as [18]:

(7) $$A/\mathrm{Ts}=\mathrm{EXP}\left[\left(\mathrm{zs}-\mathrm{ws}\right)/\mathrm{us}\right]$$

Here, A/T_s_ is the equivalence ratio calculated based on richness, and z_s_, w_s,_ and u_s_ were all from the STAR model.

(8) $$A/\mathrm{Tp}=\mathrm{EXP}\left[\left(\mathrm{zp}-\mathrm{wp}\right)/\mathrm{up}\right]$$

Here, A/T_p_ is extended from A/T_s_ and is the equivalence ratio calculated based on phylogenetic diversity. The z_p_, w_p,_ and u_p_ were all from the PTAR model. As an example, a ratio of 4 m^2^/month implies that the rate of temporal scaling in an area of 4 m^2^ is equivalent to the rate of spatial scaling over one month. These ratios enable the comparison of rates of spatial and temporal scaling in STAR and PTAR models while accounting for the interaction term [6].

### 4.7 Estimation of Bacterial rrn Copy Number

The *rrn* copy numbers for bacterial OTUs were estimated based on the *rrn*DB database (version 5.4, https://rrndb.umms.med.umich.edu/) [37]. Each OTU was matched with the database starting from the lowest rank. For OTUs with available child taxon matches, the mean *rrn* copy number of all the child taxa was used; otherwise, higher rank matches were searched, and the mean *rrn* copy number of the parent taxa for that OTU was assigned. For each sample, the community‐level *rrn* copy number, a community‐level aggregate trait value, was calculated as the mean of the estimated *rrn* copy number, weighted by the relative abundance for each OTU using equation (9) as follows:

(9) $$Community-levelrrncopynumber=\frac{\sum_{i=1}^{N}Si}{\sum_{i=1}^{N}\frac{Si}{ni}}$$

where N is the number of OTUs in a sample, *Si* is the sequence abundance of OTU*i*, and *ni* is the estimated *rrn* copy number of OTU*i*.

### 4.8 Comparison of STRs and PTRs Across Different Groups of Organisms

To obtain general insights into the spatiotemporal scaling of biodiversity across different organisms, the STAR exponents in this study were compared with all available published STAR data. Briefly, STAR was used as a keyword to search all the available literature about plants, animals, and microorganisms. After duplicates were removed, data sets were selected according to the description in the literature or were directly provided by the authors. Finally, the STAR exponents obtained from all published data sets were compared with those from our study. However, because species definition, generation time, and diversity of microbial communities are greatly different from the communities of plants and animals, detailed exact comparisons would be especially difficult across different studies [72]. Therefore, in this study, only coarse‐level comparisons were made among different types of organisms.

### 4.9 Statistical Analyses

We conducted Principal Component Analysis (PCA) using Euclidean distances to visualize differences in soil properties among different plots, utilizing the Vegan package in R v4.2.1. The Analysis of Similarities (ANOSIM) was performed to examine the significance. Differences in soil physicochemical properties between plots at each site were tested using linear mixed models, with the time point set as a random effect. To assess the spatial heterogeneity of each soil physicochemical property, we calculated the coefficient of variation (CV; standard deviation/mean) at each time point. For assessing the temporal heterogeneity of each soil physicochemical property, we calculated the detrended coefficient of variation (residuals of linear regression/mean) for each sampling position. Furthermore, we calculated Cohen's d as an estimate of bioenergy cropping (or soil texture) effect sizes on the spatial/temporal heterogeneity of soil physicochemical properties from different treatments [i.e., bioenergy cropping (or clay loam)] by comparing them against the common control [i.e., fallow (or sandy loam)].

To minimize the potential bias caused by spatiotemporal autocorrelation, the significance of the STAR and PTAR models was evaluated using the permutation test (rand = 1,000). Specifically, we randomly permuted the temporal and spatial order, refit the model for each permutation, and constructed a null distribution of R^2^; the significance of the model was then assessed by evaluating how extreme the observed R^2^ was relative to this null distribution. To compare exponent differences between the STAR and PTAR models, we used permutation tests (rand = 1,000) by evaluating whether the observed differences between STAR and PTAR exponents fell within the null distribution generated from differences between randomly permuted STAR and PTAR exponents. Permutation tests were implemented using a restricted block permutation. Temporally adjacent observations and spatially neighboring nested areas were grouped into blocks of three consecutive scales, and blocks were permuted as intact units to preserve local dependence. Differences in STAR and PTAR model exponents among bacterial phyla were tested using ANOVA. Differences in rrn copy number among bacterial phyla were assessed with a Kruskal–Wallis test because sample sizes were unequal. Correlations between phylum‐level STAR and PTAR exponents and the corresponding rrn copy numbers were evaluated using Spearman correlation analyses.

We also tested whether the exponents in the STARs or the exponents in the PTARs were significantly different between any two treatments and between any two sites using a linear mixed model. Prior to this, we obtained many STAR and PTAR models for each treatment at each site by using the moving window method. During this process, we traversed all possible window sizes (from 5 to 14 months; that is, time length) and set the step as 1, so we can get 13 STAR/PTAR models for window size 5, 12 STAR/PTAR models for window size 6, 11 STAR/PTAR models for window size 7, and so on. The total effect size of bioenergy cropping on the exponents of the STAR and PTAR models was estimated with a linear mixed model by using the *lme* function. The formula of the linear mixed model was as follows:

(10) $$\begin{array}{ccc} & & Y\;∼\;\mathrm{Measure}\;+\left(1|\mathrm{Windowsize}/\mathrm{Site}/\mathrm{PlotID}\right)+\mathrm{corAR}1\\ & & \left(\mathrm{form}=∼\mathrm{ystart}|\mathrm{Windowsize}/\mathrm{Site}/\mathrm{PlotID}\right) \end{array}$$

where window size, site, and plot ID were treated as random factors. The random effect structure (1|Windowsize/Site/PlotID) was specified to account for the hierarchical structure of the data, thereby allowing each level of the hierarchy to vary in its baseline response. The term corAR1 was used to account for temporal autocorrelation, with ystart representing the starting time point of each window size.

The total effect size of soil texture on the exponents of the STAR and PTAR models was estimated with a linear mixed model by using the *lme* function. The formula of the linear mixed models was as follows:

(11) $$\begin{array}{ccc} & & Y∼\;\mathrm{Soil}\;\mathrm{Type}\;+\left(1|\mathrm{Windowsize}/\mathrm{Measure}/\mathrm{PlotID}\right)\\ & & +\;\mathrm{corAR}1\left(\mathrm{form}=∼\mathrm{ystart}|\mathrm{Windowsize}/\mathrm{Measure}/\mathrm{PlotID}\right) \end{array}$$

where window size, measure, and plot ID were treated as random factors. The term corAR1 was used to account for temporal autocorrelation, with ystart representing the starting time point of each window size.

For the effect size of bioenergy cropping at each site (clay and sandy loam sites) and that of soil texture in each treatment (bioenergy cropping and fallow), the time window size was considered as a random factor in the linear mixed model, while temporal autocorrelation among window sizes was accounted for using a corAR1(form = ∼ ystart|Windowsize) structure. Additionally, the ln‐response ratio [ln(treat_mean_/control_mean_)] of bioenergy cropping (or soil texture) and its variance were also calculated. The significant difference in slopes from SAR, PAR, STR, and PTR among the four plots was examined by the permutation test (rand = 1,000). The effects of bioenergy cropping (or soil texture) on taxonomic and phylogenetic spatiotemporal scaling rates among different bacterial phyla are presented as the relative changes of (bioenergy cropping − fallow)/fallow [or (clay loam − sandy loam)/sandy loam]. The significance was examined by the permutation test (rand = 1,000). We used linear mixed models to examine relationships between spatial and temporal scaling rates and both community stability (community stability was quantified as the inverse of the coefficient of variation of bacterial richness across the time series) and the ecosystem function (CH_4_ fluxes).

A Mantel test was performed to determine the correlation between soil properties and bacterial community composition. The variation partitioning analysis was performed to determine the contributions of groups of soil physicochemical properties to total variations in soil bacterial community composition. Additionally, the correlations between the heterogeneity of soil properties and STAR (or PTAR) exponents were examined by the linear regression model (for bacterial community) and the Spearman correlation method (for bacterial phyla; the p values were adjusted by the FDR method).

The neutral community model used in this study predicts the relationship between taxon frequency and abundance within a set of local communities [73]. In this model, the migration rate (m) represents the dispersal capacity, with higher values indicating less dispersal limitation. Here, this rate was calculated using the mean relative abundance of observed OTUs. For each timepoint, we calculated a neutral community model, so finally we got 17 neutral community models for each treatment at each site. The effect size of bioenergy cropping on the m values at each site was estimated with a linear mixed model, with timepoint being considered as a random factor. All the analyses were performed in the R software v.4.1.2.

## Conflicts of Interest

The authors declare no conflicts of interest.

## Code Availability Statement

The codes have been deposited to GitHub (https://github.com/2022310233/STAR).

## Supporting information




**Supporting File**: advs74821‐sup‐0001‐SuppMat.docx.

## Data Availability

The data that support the findings of this study are openly available in NCBI Sequence Read Archive at https://www.ncbi.nlm.nih.gov/sra/ PRJNA6896656, reference number 6896656.
