# Supplementary material for: Bioenergy Cropping Reduces the Spatiotemporal Scaling of Soil Bacterial Biodiversity
Source: Adv Sci (Weinh). 2026 Mar 17;13(29):e18964. doi: 10.1002/advs.202518964 (PMC13205762; doi:10.1002/advs.202518964)
Supplement: Supplementary file 1 — Supporting File: advs74821‐sup‐0001‐SuppMat.docx. [file ADVS-13-e18964-s001.docx]

**Supporting text**

***S1. The SAR and PAR of the soil bacterial community***

In ecology, the relationship between species richness and area is often described by the power-law equation S = cA^zs1^, or its logarithmic equation. We analyzed SARs for soil bacterial communities across different plots. In addition, incorporating phylogenetic-based patterns is essential for effective conservation planning, optimizing evolutionary history preservation, and testing biodiversity theories^[1, 2]^. Therefore, we generated phylogenetic analogs of SAR models (PARs). Similarly, PARs are described by the power-law equation PD = cA^zp1^ or its logarithmic equation. Here, z-values represent the slopes of SARs (z_s1_) and PARs (z_p1_) in log-log space, indicating spatial scaling rates. We found a strong fit of soil bacterial diversities to the logarithmic model in both fallow and bioenergy cropping (switchgrass) plots at the sandy loam and clay loam sites based on species richness and phylogenetic diversity (p < 0.001; Figure S1a-b, Supporting Information), indicating robust SARs and PARs in soil bacterial communities. Consistent with our findings, significant diversity scaling relationships have been reported for indigenous bacteria across various ecosystems, such as water-filled tree holes^[3, 4]^, salt marshes^[5]^, grasslands^[6, 7]^, and islands^[8]^, suggesting that SARs are universal for microorganisms across various environments. The estimated z_s1_ values of the bacterial community ranged from 0.092 to 0.101 (Figure S1a-b, Supporting Information), falling within the range of microbial scaling rates (0.002 < z < 0.260) compiled by previous research^[9]^. As a piece of evidence of faster taxonomic scaling compared to phylogenetic scaling, we found that the z_s1_ values (0.092-0.101) were higher than the z_p_ values (0.088-0.093), indicating that SARs were phylogenetically conserved. Moreover, the z-values exhibited an inverse relationship with the length of time (Figure S1c-d, Supporting Information; p < 0.001), with bioenergy cropping plots significantly reducing their slopes compared to fallow plots (p < 0.050, examined by permutation test).

***S2. The STR and PTR of soil bacterial communities***

Through monthly sampling, we analyzed STRs and PTRs for soil bacterial communities across different plots by using the nested method^[10]^. The w-values represent the slopes of STRs (w_s1_) and PTRs (w_p1_) in a log-log space, indicating temporal scaling rates. We observed significant STRs and PTRs in all four plots (Figure S2a-b, Supporting Information; p < 0.001). Consistent with our findings, significant STR patterns have been reported for bacteria across various systems, such as water-filled tree holes^[4]^, leaf surface^[11]^, bioreactors^[12]^, and grasslands^[6]^. The resulting w_s1_ values ranged from 0.255 to 0.332, within the typical microbial scaling rate range (0.240 < w < 0.610)^[4, 13]^, while w_p1_ values ranged from 0.229 to 0.313. However, there were few studies on the phylogenetic diversity-based scaling of bacterial communities. Lower w_p1_ values compared to w_s_ values indicated faster taxonomic scaling than phylogenetic scaling over time. Moreover, the w-values exhibited an inverse relationship with plot area (Figure S2c-d, Supporting Information; p < 0.001), with bioenergy cropping plots significantly reducing their slopes compared to fallow plots (p < 0.050, examined by permutation test). Additionally, we noticed that the w-values were considerably larger than z-values, which was consistent with the results of STAR and PTAR (Figures S1 and S2, Supporting Information). This finding indicated that bacterial diversity accumulated faster over time than across space.

***S3. Changes of SAR, PAR, STR, and PTR slopes across different microbial lineages***

We observed strong spatial and temporal diversity patterns across bacterial lineages (Table S2, Supporting Information). The spatial and temporal scaling rates varied considerably across different phyla (z_s1_ = 0.072-0.165, z_p1_ = 0.055-0.132, w_s1_ = 0.266-0.637, w_p1_ = 0.182-0.419), with *Acidobacteria* and *Actinobacteria* displaying the lowest scaling rates. *Acidobacteria* and *Actinobacteria* are comprised of many oligotrophic bacterial taxa^[14]^ (also supported by the low rrn copy numbers; Figure S6b, Supporting Information), suggesting that their slow growth rates may contribute to the lower scaling rates.

***S4. Effects of bioenergy cropping on bacterial SAR, PAR, STR, and PTR***

Interestingly, both z_s1_ and z_p1_ values in bioenergy cropping plots were lower than those in fallow plots, with a decrease of 2.0%-5.2% (p = 0.067, examined by permutation test; Figure S7a, Supporting Information). The z_s1_ and z_p1_ values were higher at the clay loam site than at the sandy loam sites (p > 0.050, examined by permutation test; Figure S7b, Supporting Information). Additionally, both w_s1_ and w_p1_ values in bioenergy cropping plots were significantly lower than those in fallow plots (p < 0.001, examined by permutation test; decreased by 6.9%-11.5%; Figure S7a, Supporting Information). Moreover, the w_s1_ and w_p1_ values were significantly higher at the clay loam site than the sandy loam site (p < 0.001, examined by permutation test; Figure S7b, Supporting Information).

Consistent with the overall bacterial community, bioenergy cropping decreased the spatial and temporal scaling rates of most phyla compared to fallow plots. However, some bacterial phyla, such as *Firmicutes*, exhibited an increasing trend in those values after bioenergy cropping (Table S2, Supporting Information). This suggests different response mechanisms of various bacterial taxa to bioenergy cropping. The increased nutrient levels under bioenergy cropping (Table S4, Supporting Information) may promote the scaling of *Firmicutes*, which had high rrn copy numbers (Figure S6b, Supporting Information). Similar to the overall bacterial community, the scaling rates of most bacterial phyla were higher at the clay loam site than the sandy loam site (Table S2, Supporting Information). Additionally, we also observed lower z_p1_ and w_p1_ values compared to z_s1_ and w_s1_ values for almost all phyla in the four plots (Table S2, Supporting Information).

***S5. Mechanisms underlying changed bacterial spatiotemporal scaling***

The observed changes in microbial diversity patterns could be caused by various soil properties. We observed strong disparities in soil properties (Soil TC, TN, P, NO_3_^-^, NH_4_^+^, pH, and soil moisture) between the sandy loam and clay loam sites through principal component analyses (Figure S12a, Supporting Information). There were significant differences in these soil properties between the bioenergy cropping and fallow plots, regardless of soil textures (Figure S12b, Supporting Information; p < 0.001 by ANOSIM). Generally, the soil at the clay loam site exhibited better nutrient conditions (TC, TN, NO_3_^-^, and NH_4_^+^) compared to the sandy loam site (Table S4, Supporting Information; p < 0.001, determined by LMM). Bioenergy cropping significantly enhanced the concentration of soil properties (except for NO_3_^-^; p < 0.050, determined by LMM) at the sandy loam site, while significantly increasing soil NO_3_^-^ concentration at the clay loam site (p < 0.050, determined by LMM; Table S4, Supporting Information). Compared with fallow plots, bioenergy cropping decreased the spatial variability of most soil properties at clay loam and sandy loam sites, though there was no significant difference (p > 0.005; examined by Turkey HSD test; Figure S10, Supporting Information). Bioenergy cropping significantly decreased the temporal variability of soil moisture at the clay loam site (p < 0.050; examined by Turkey HSD test; Figure S10, Supporting Information).

Bacterial community compositions in the four plots were significantly influenced by several common soil properties, including soil pH, moisture, P, and TC (Table S6, Supporting Information). However, only small portions (10.0%-12.0%) of the variations in bacterial community composition were explained by the soil properties examined (variation partitioning analyses; Figure S12c, Supporting Information). A substantial portion of community variations (88.0%-90.0%) remained unexplained by measured soil properties. This is consistent with other studies^[6, 8]^, indicating that a substantial amount of microbial community composition variation remained unexplained by measured environmental variables. A significant portion of this unexplained variation may stem from local effects of unmeasured biotic (e.g., competition and trophic interactions)^[15]^ or abiotic (e.g., the heterogeneity of soil properties, O_2_ levels within soil aggregates and labile C pools)^[16]^ factors, or stochastic processes^[6, 17]^.

Given that a previous study reported that the scaling of soil bacteria was determined by environmental heterogeneity^[18]^, we fitted the variabilities of soil properties with bacterial community spatial and temporal scaling rates by using linear regression models. Soil P variability was significantly and positively correlated with the richness-based spatial and temporal scaling rates (p < 0.050; Figure S12d, Supporting Information), while there was no significant correlation between the variabilities of soil properties and the phylogenetic-based spatial and temporal scaling rates. Thus, the decrease in soil heterogeneity caused by bioenergy cropping can lead to the loss of ecological niches, thereby reducing the scaling of bacterial communities. Moreover, the bacterial community in bioenergy cropping plots may experience selection due to the influence of root exudates, leading to more specialized microbial taxa such as associative nitrogen-fixing bacteria^[19]^. This specialization can also reduce the scaling rates of bacterial communities.

**
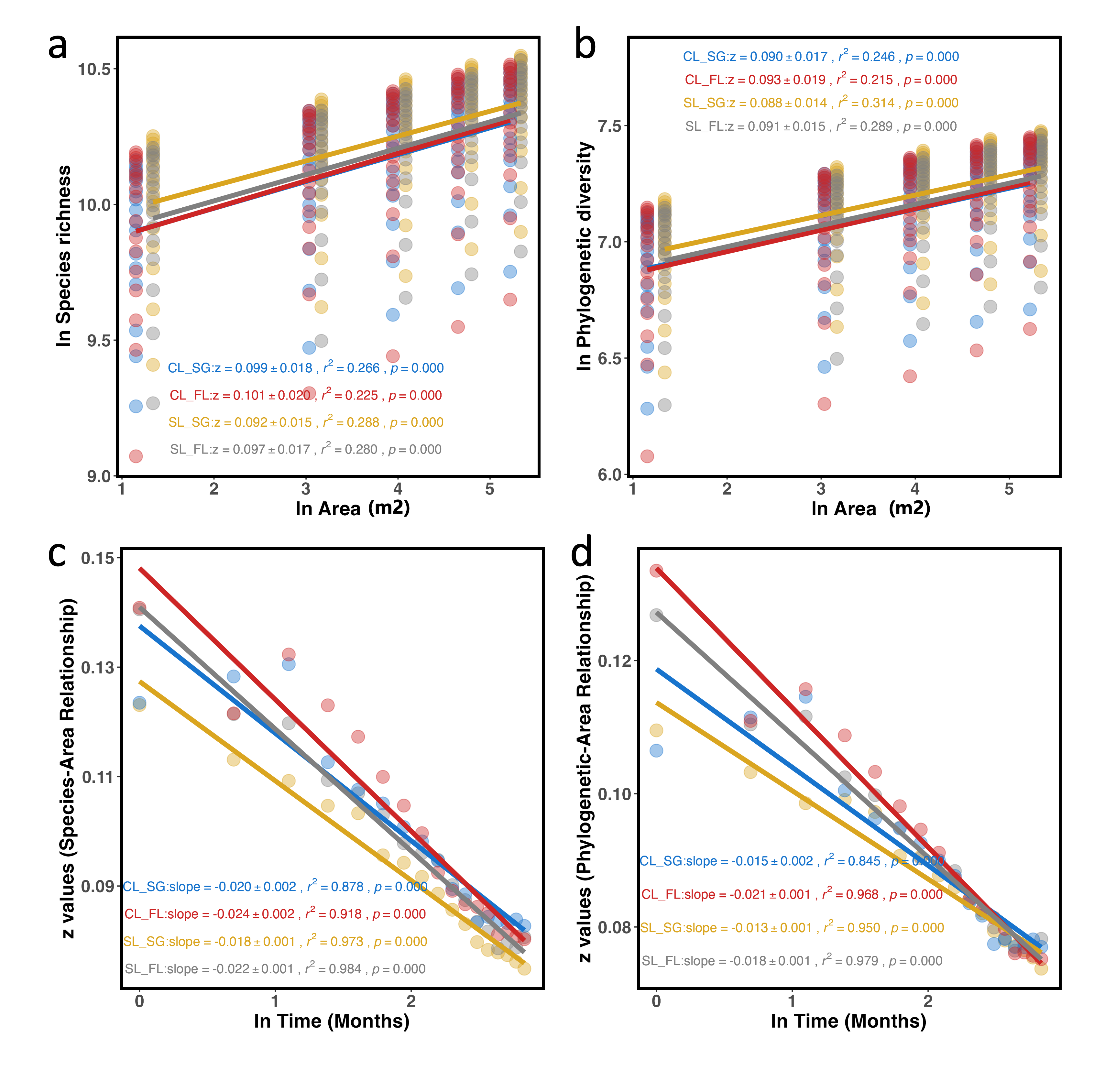
**

**Figure S1 The spatial patterns of bacterial diversity. a**, species-area relationships of the four plots. **b**, phylogenetic-area relationships of the four plots. **c**, the relationship between richness-based z-values (z_s1_) and time length. **d**, the relationship between phylogenetic-based z-values (z_p1_) and time length. The p-values were adjusted by the FDR method. SG: bioenergy cropping (Switchgrass), FL: fallow, SL: sandy loam, CL: clay loam.

**
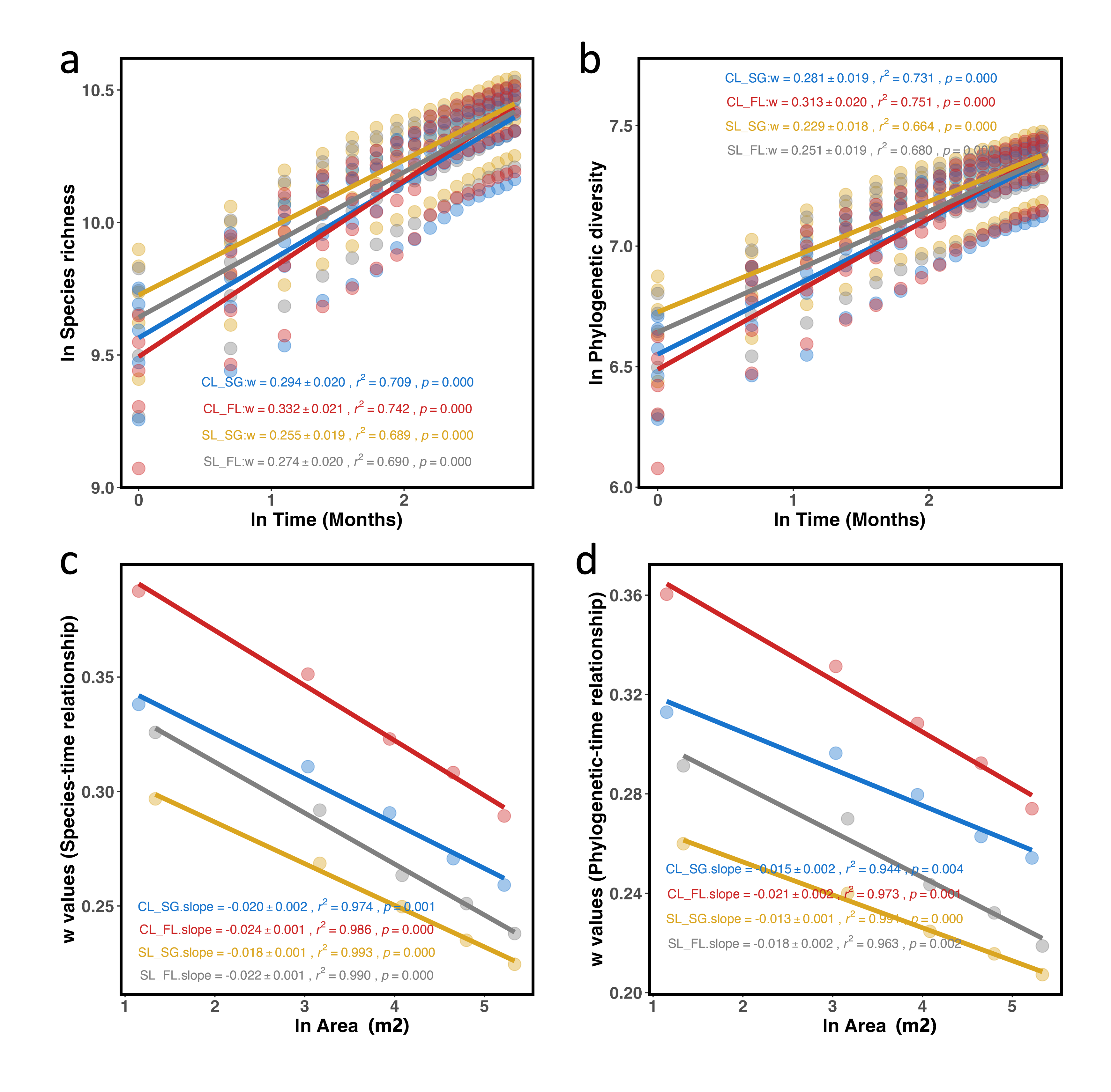
**

**Figure S2 The temporal patterns of bacterial diversity. a**, species-time relationships of the four plots. **b**, phylogenetic-time relationships of the four plots. **c**, the relationship between richness-based w-values (w_s1_) and area. **d**, the relationship between phylogenetic-based w-values (w_p1_) and area. The significance was examined by a permutation test. SG: bioenergy cropping (Switchgrass), FL: fallow, SL: sandy loam, CL: clay loam.

**
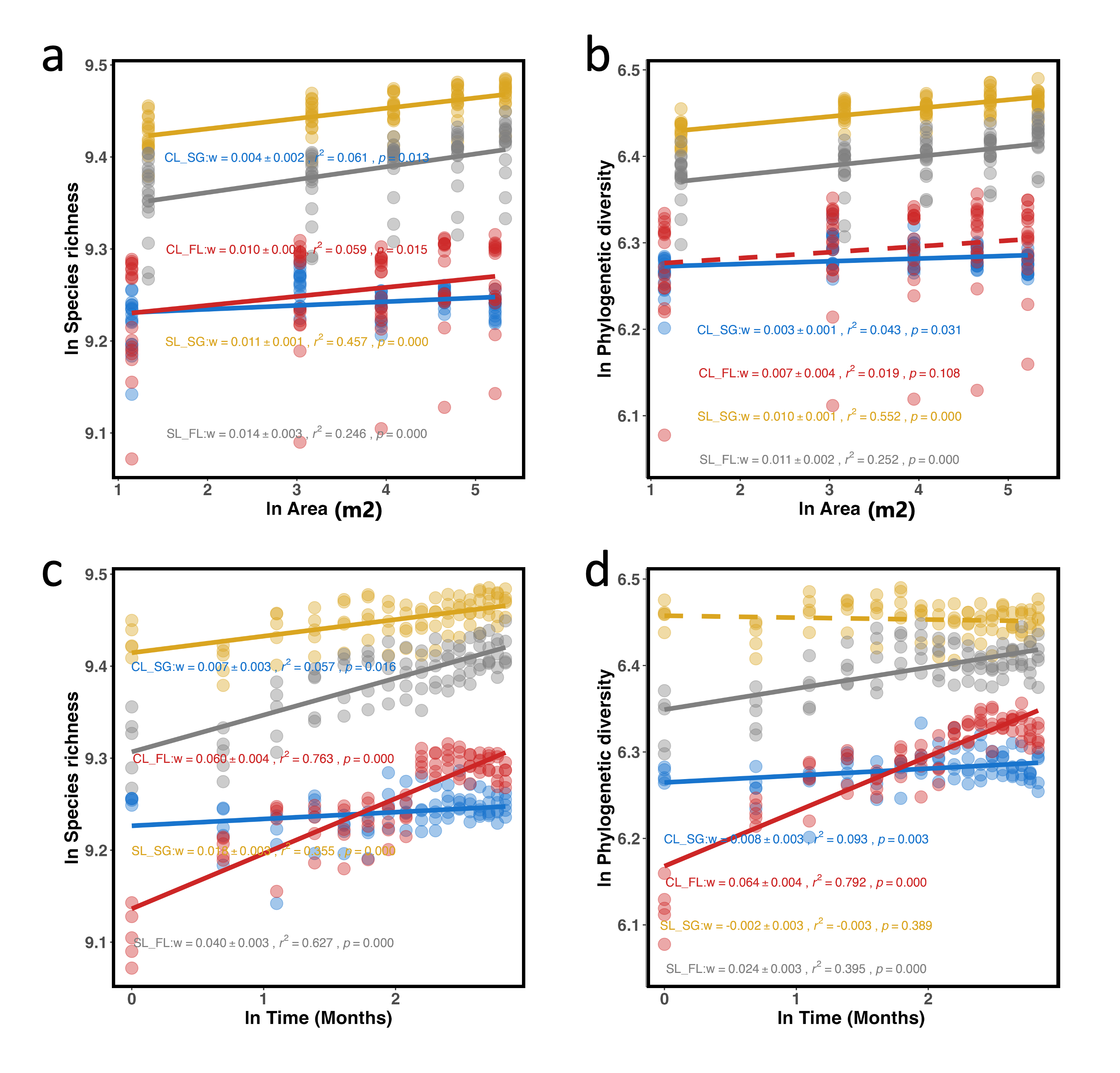
**

**Figure S3 The temporal and spatial patterns of bacterial diversity when standardizing the total number of bacterial sequences for each spatial or temporal scale.** SG: bioenergy cropping (Switchgrass), FL: fallow, SL: sandy loam, CL: clay loam. **a**, species- area relationships of the four plots. **b**, phylogenetic-area relationships of the four plots. **c**, species-time relationships of the four plots. **d**, phylogenetic-time relationships of the four plots.


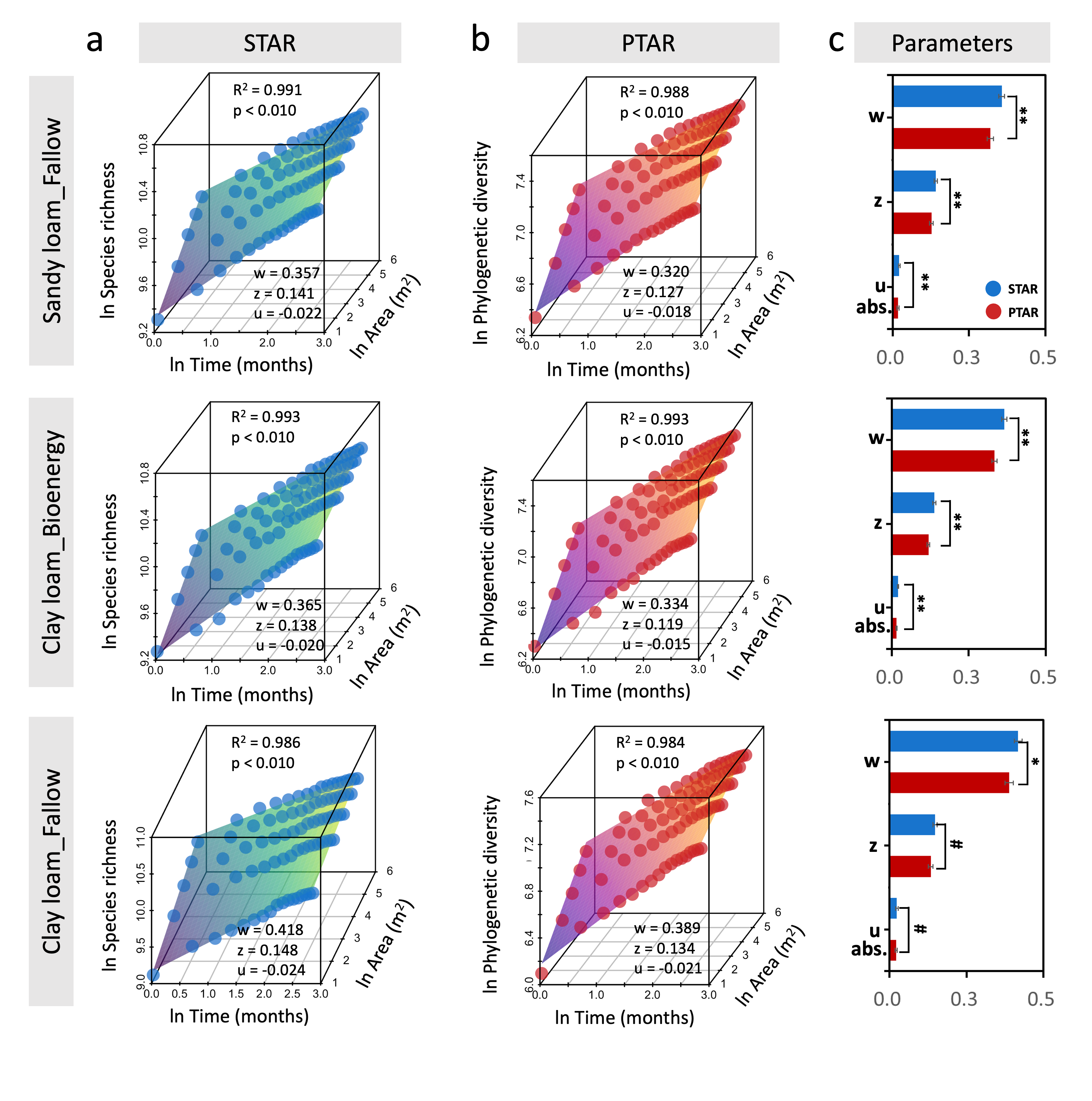


**Figure S4 Bacterial diversity as a function of time, area, and a time–area interaction term. a**, The species-time-area relationships (STRAs) in soil bacterial communities in bioenergy cropping and fallow plots at sandy loam and clay loam sites. **b**, The phylogenetic-time-area relationships (PTRAs) in soil bacterial communities in bioenergy cropping and fallow plots at sandy loam and clay loam sites. **c**, The difference in bacterial spatiotemporal scaling rates between taxonomic and phylogenetic diversity. The parameters of both the STAR and PTAR models are significant (p < 0.010). The significance was examined by the permutation test (rand = 1,000). *** p < 0.001; ** p < 0.010; * p < 0.050. # p < 0.1; abs: the absolute value.


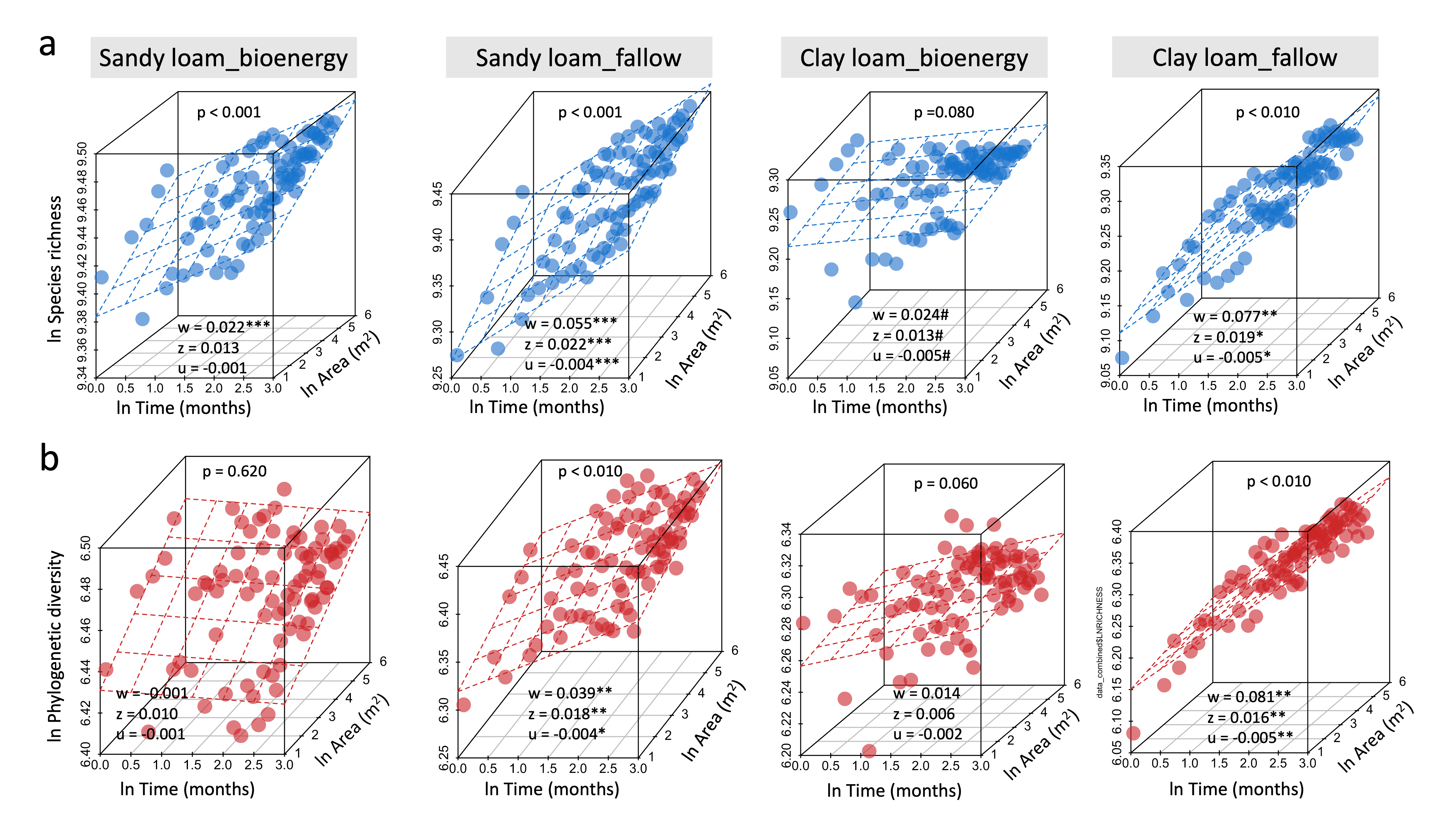


**Figure S5 The negative sampling test for Species-time-area relationship (STAR) and Phylogenetic-time-area relationship (PTAR). a**, STAR. **b**, PTAR. The significance was examined by the permutation test (rand = 1,000). *** p < 0.001; ** p < 0.010; * p < 0.050. # p < 0.100.

**
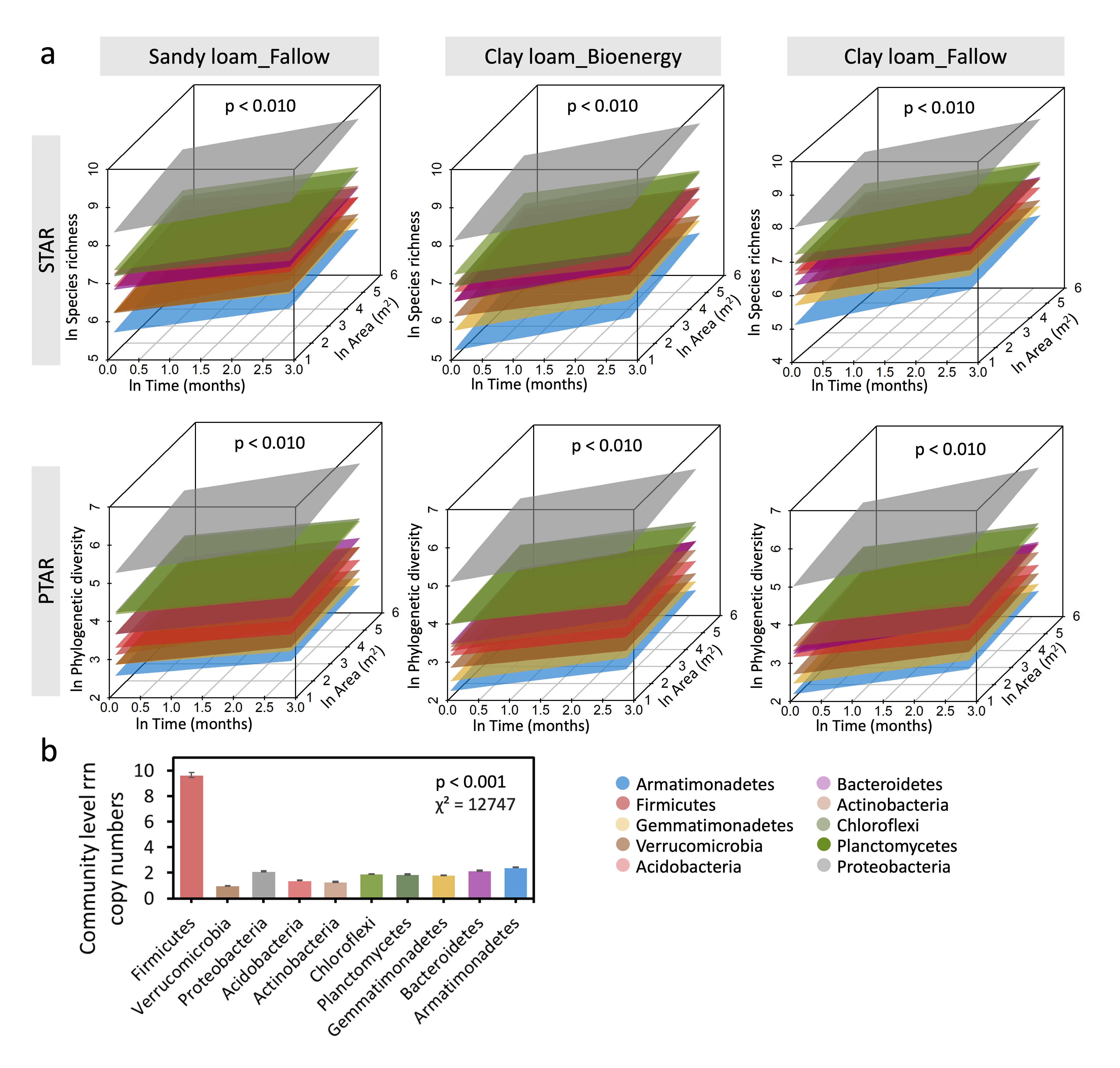
**

**Figure S6 The spatiotemporal scaling patterns in different bacterial phyla. a**, The species-time-area relationships (STARs) and phylogenetic-time-area relationships (PTARs) in different bacterial phyla in bioenergy cropping and fallow plots at sandy loam and clay loam sites. The significance was examined by the permutation test (rand = 1,000). **b**, The community-level rrn copy numbers of major common bacterial phyla. Differences in rrn copy number among bacterial phyla were assessed with a Kruskal–Wallis test because sample sizes were unequal.

**
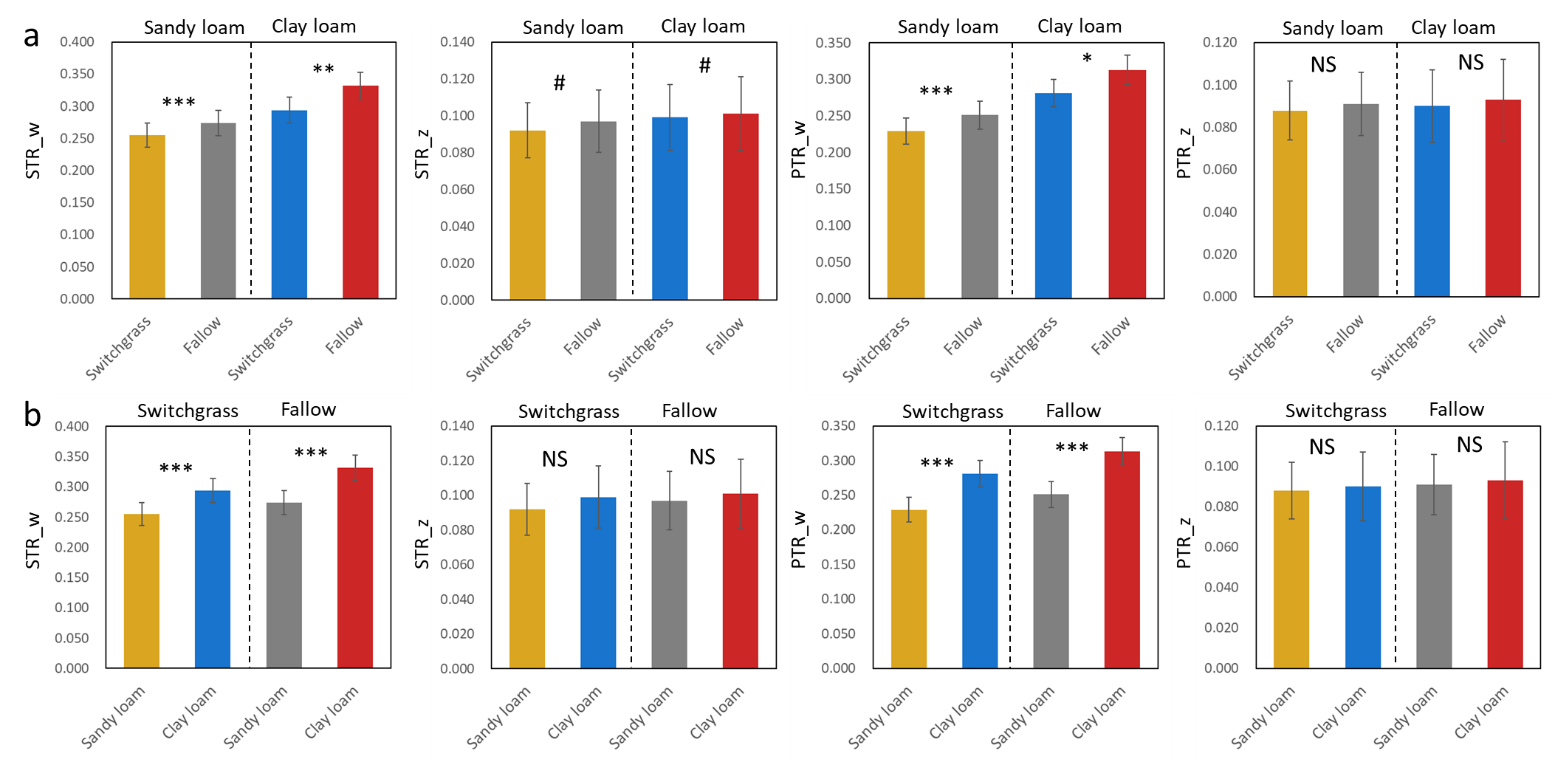
**

**Figure S7 Permutation test of slope for the single-variable model.** **a**, The difference in bacterial spatiotemporal scaling rates between bioenergy cropping (switchgrass) and fallow plots. **b**, The difference in bacterial spatiotemporal scaling rates between sandy loam and clay loam soils. The significance was examined by the permutation test (rand = 1,000). *** p < 0.001; ** p < 0.010; * p < 0.050; # p < 0.100; NS: no significance.


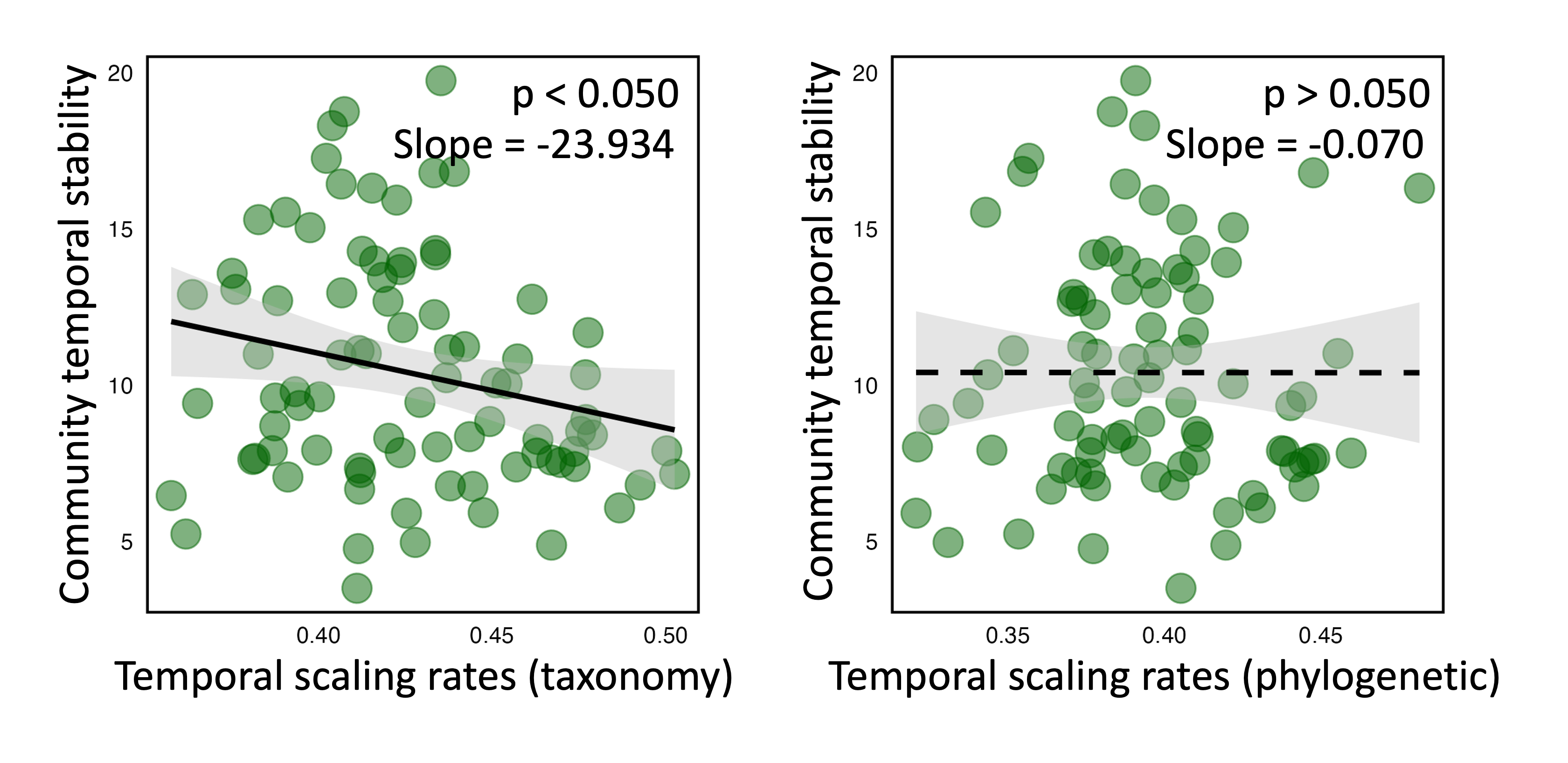


**Figure S8 Correlations between bacterial temporal scaling rates and community temporal stability.** The significance was tested by the linear mixed model.


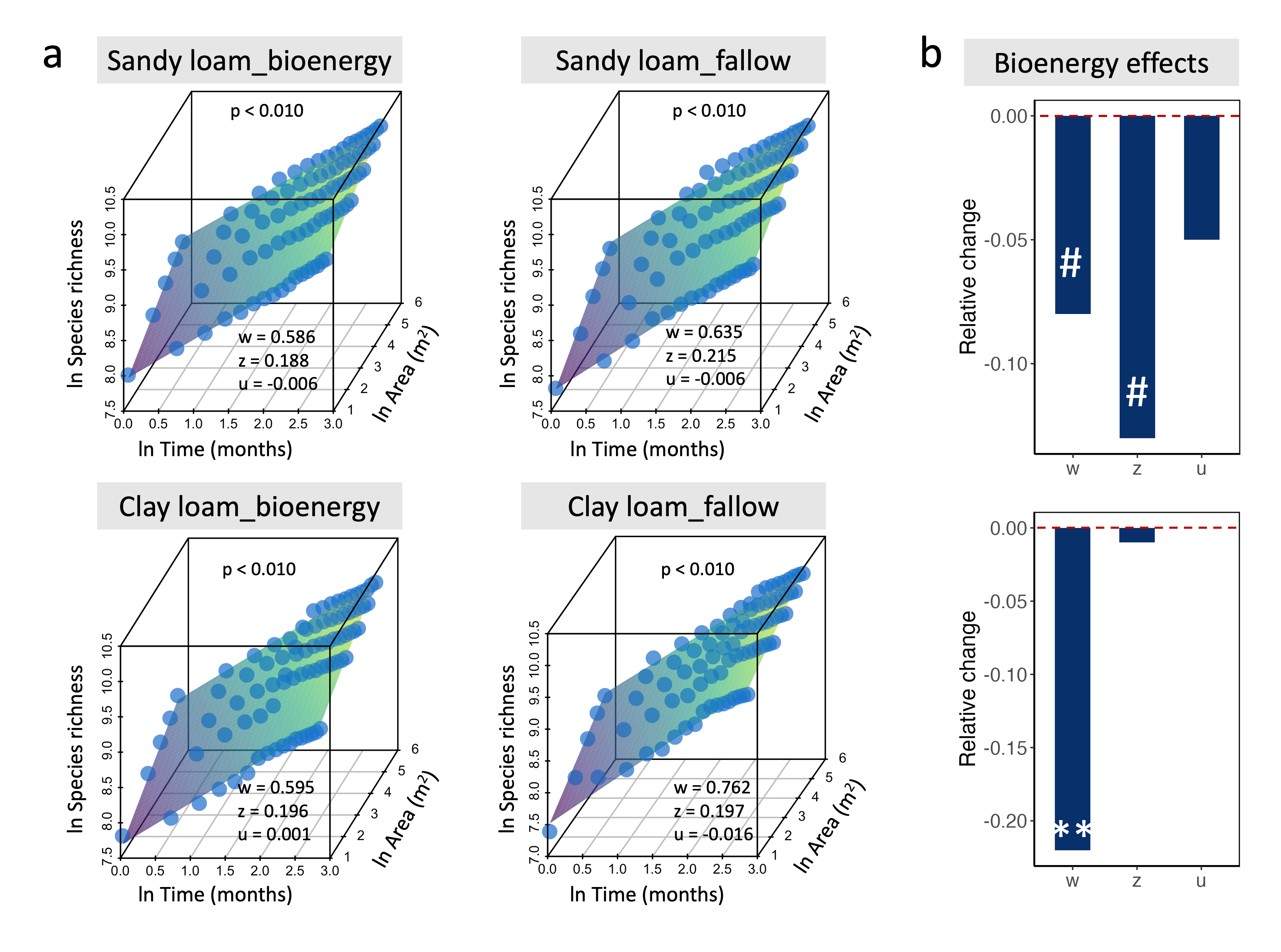


**Figure S9 The DADA2-based bacterial spatiotemporal scaling pattern.** **a**, Species-Time-Area relationship. The significance was examined by the permutation test (rand = 1,000). **b**, The bioenergy cropping effects on the spatiotemporal scaling rates. The effects are presented as the relative changes of (bioenergy cropping-fallow)/fallow. At the clay loam site, STAR u turned positive after bioenergy cropping, and its response to the bioenergy cropping was not calculated here. The significance was examined by the permutation test (rand = 1,000). ** p < 0.010; # p < 0.100.

**
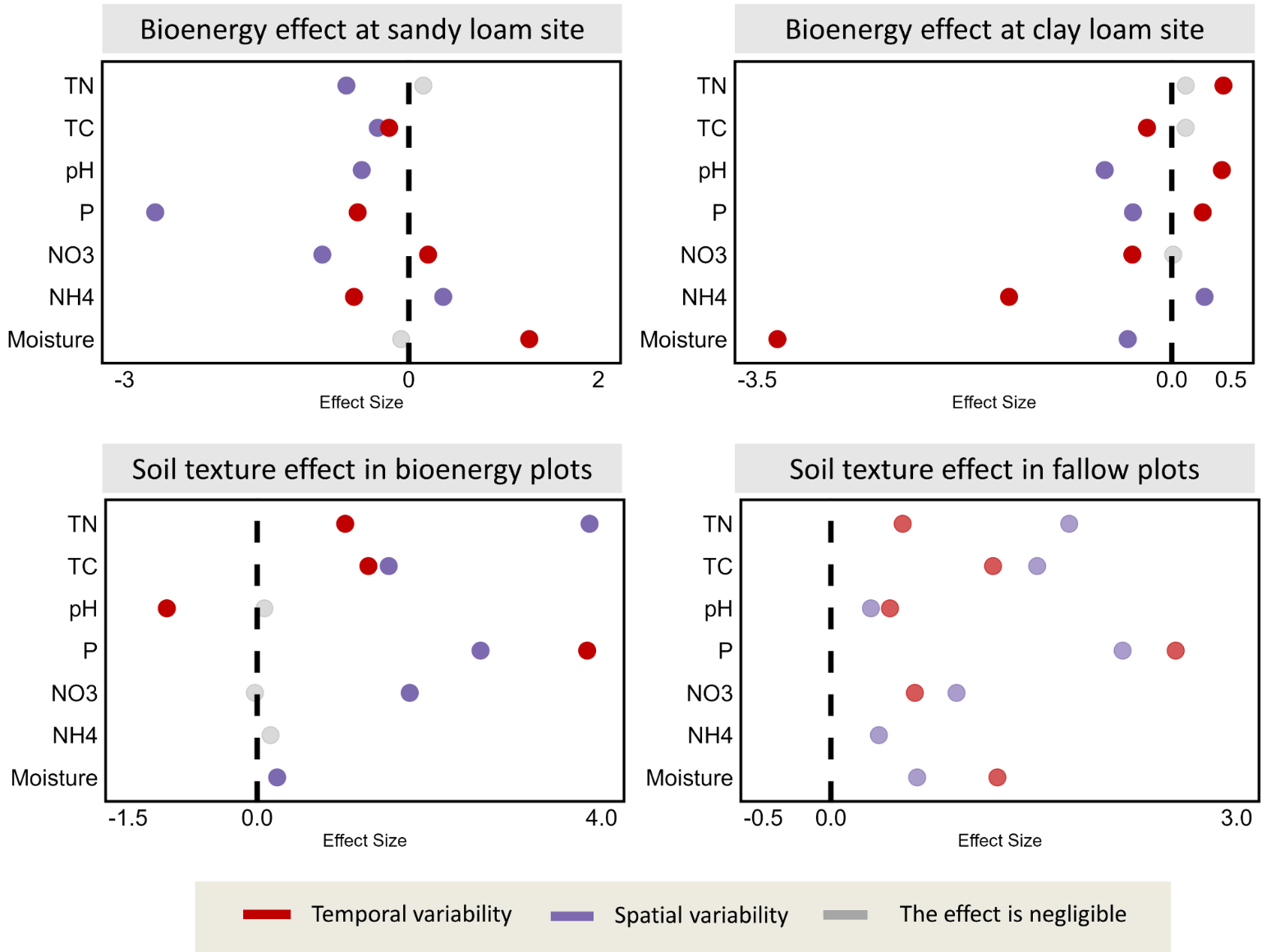
**

**Figure S10 The effect size of bioenergy and soil texture on the temporal and spatial variabilities of soil properties.** The bioenergy effect is calculated by comparing the bioenergy cropping with fallow, while the soil texture effect is calculated by comparing the clay loam with sandy loam soils. Effect size is estimated with Cohen’s d. d > 0.200, small effect; d > 0.500, medium effect; d > 0.800, large effect. A positive effect indicates a larger soil variability compared to the control treatment, with the reciprocal being true for negative effects. If the effect size is less than 0.200, it is considered negligible and colored gray.


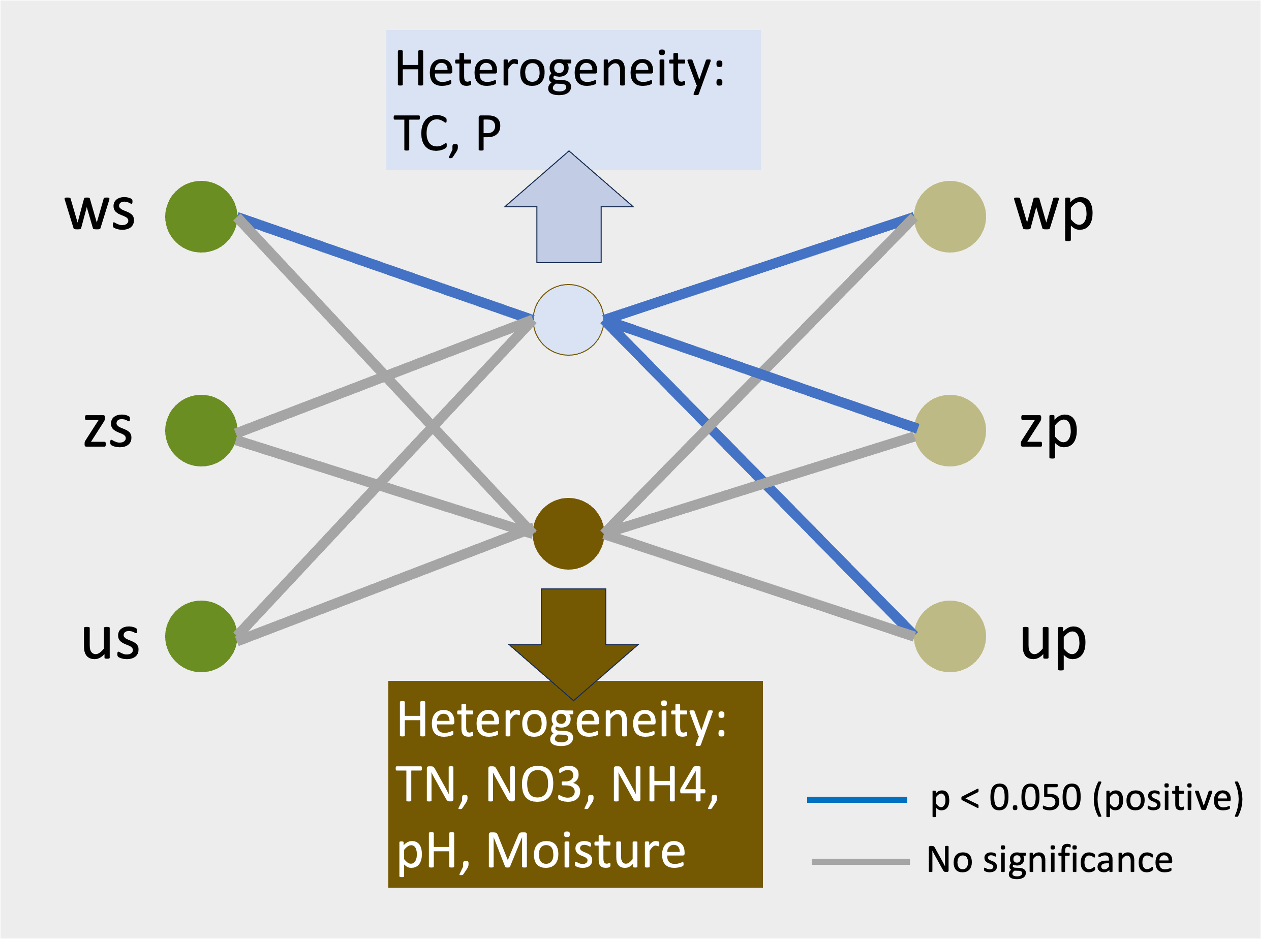


**Figure S11 Linear fitting between Spatiotemporal scaling rates and soil heterogeneity based on Euclidean distance.** The ws: taxonomic-based temporal scaling rate, zs: taxonomic-based spatial scaling rate, us: the taxonomic-based time-space interaction term. The wp: phylogenetic-based temporal scaling rate, zp: phylogenetic -based spatial scaling rate, up: the phylogenetic-based time-space interaction term.


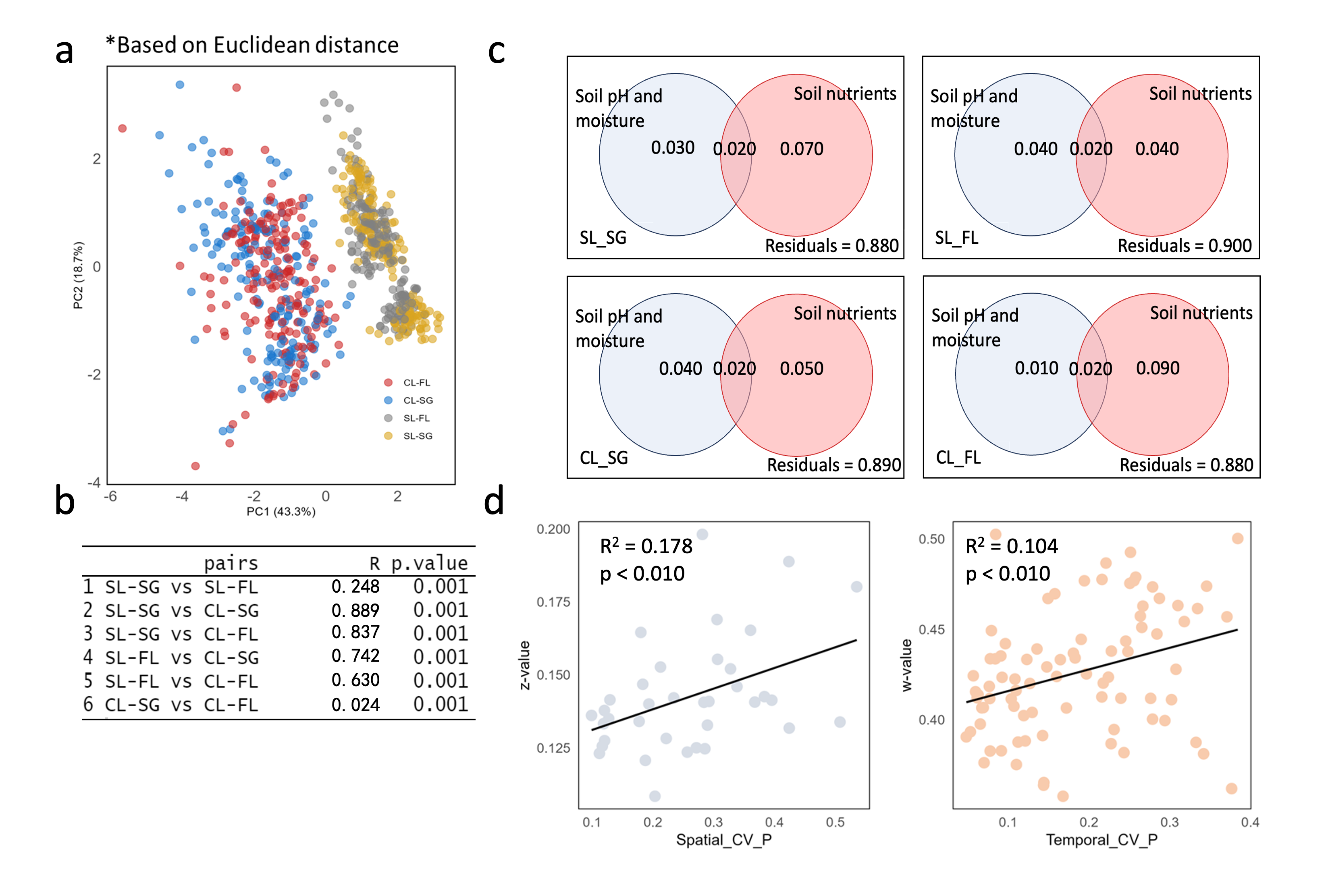


**Figure S12 The environmental factors driving the spatiotemporal scaling of bacterial community. a**, Principal Component Analysis (PCA) of soil properties among different plots based on Euclidean distances. **b**, Paired comparison of differences in soil variables between the four plots based on ANOSIM (Analysis of Similarities). **c**, The explained variance of soil properties on changes in bacterial community composition revealed by variance decomposition analysis. **d**, Correlations between spatial (zs1) and temporal scaling rates (ws1) and the variabilities of soil P. The variability (coefficient of variation; CV) was treated as the soil heterogeneity. SL: sandy loam soil; CL: clay loam soil; SG: switchgrass; FL: Fallow.


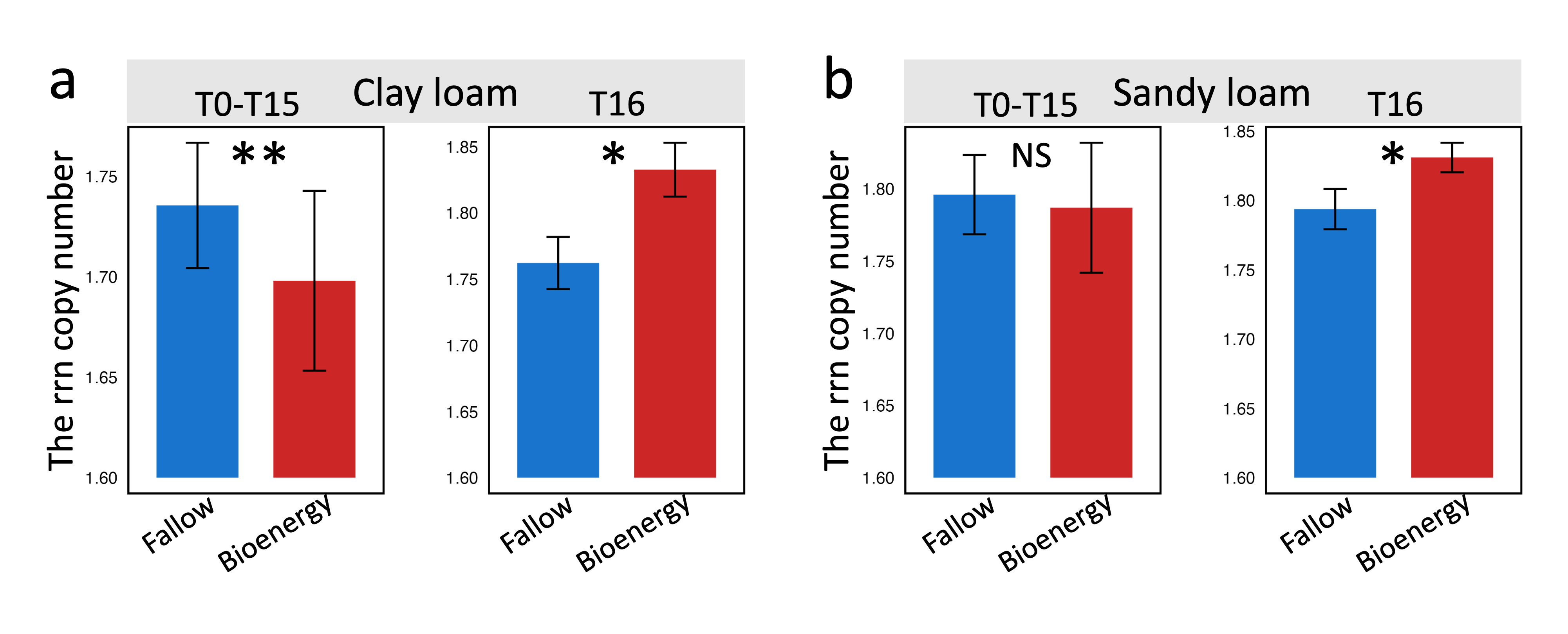


**Figure S13 Effect of bioenergy cropping on the bacterial community-level rrn copy number. a**, clay loam site. **b**, sandy loam site. The significance was tested by the linear mixed model with timepoint and sampling position being random factors (n = 336 for T0-T15, n = 21 for T16). ** p < 0.010; * p < 0.050; NS: no significance.


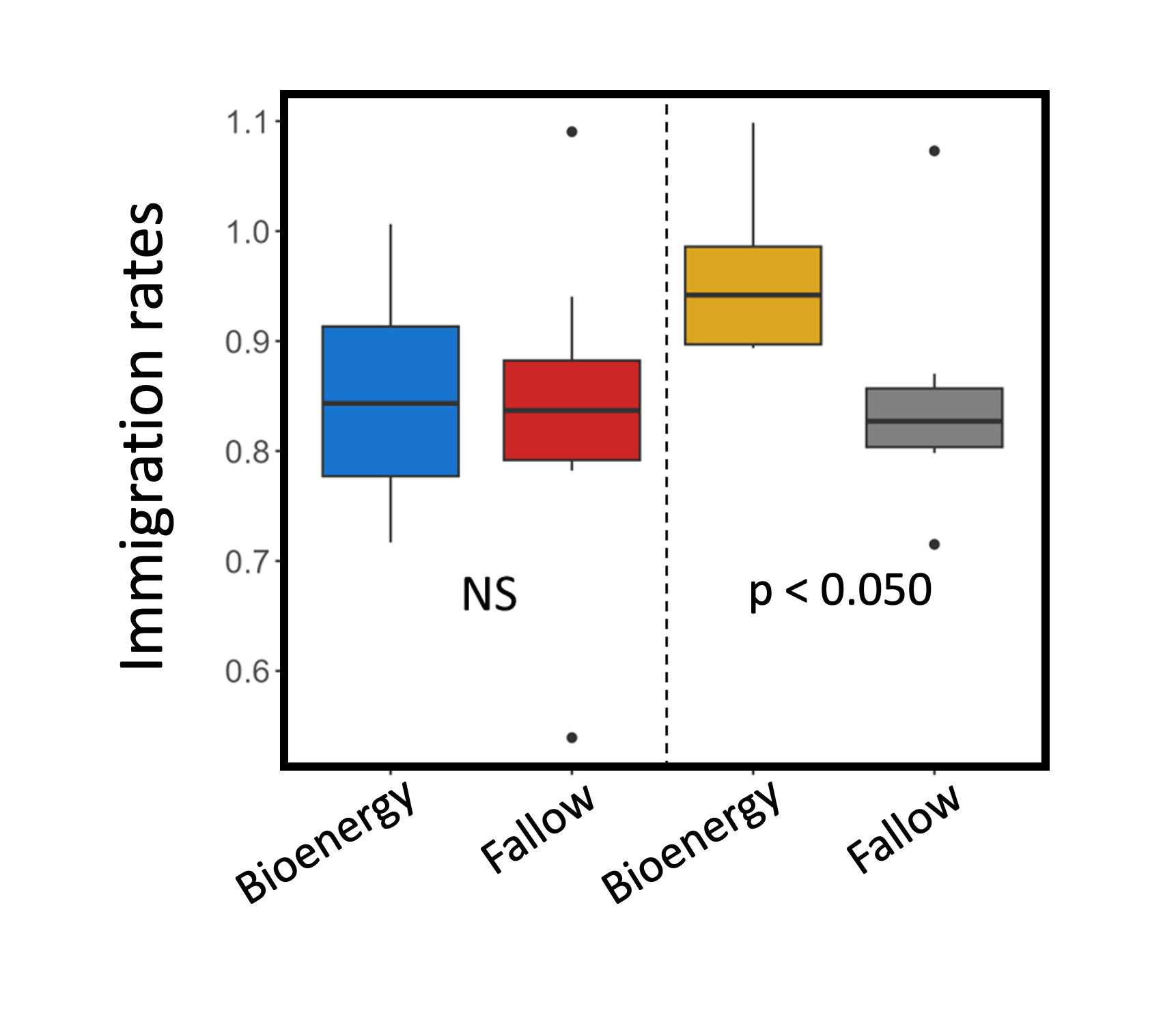


**Figure S14 Effect of bioenergy cropping on the bacterial community immigration rates.** The significance is tested by the linear mixed model with the timepoint being a random factor (n = 17). NS: no significance.


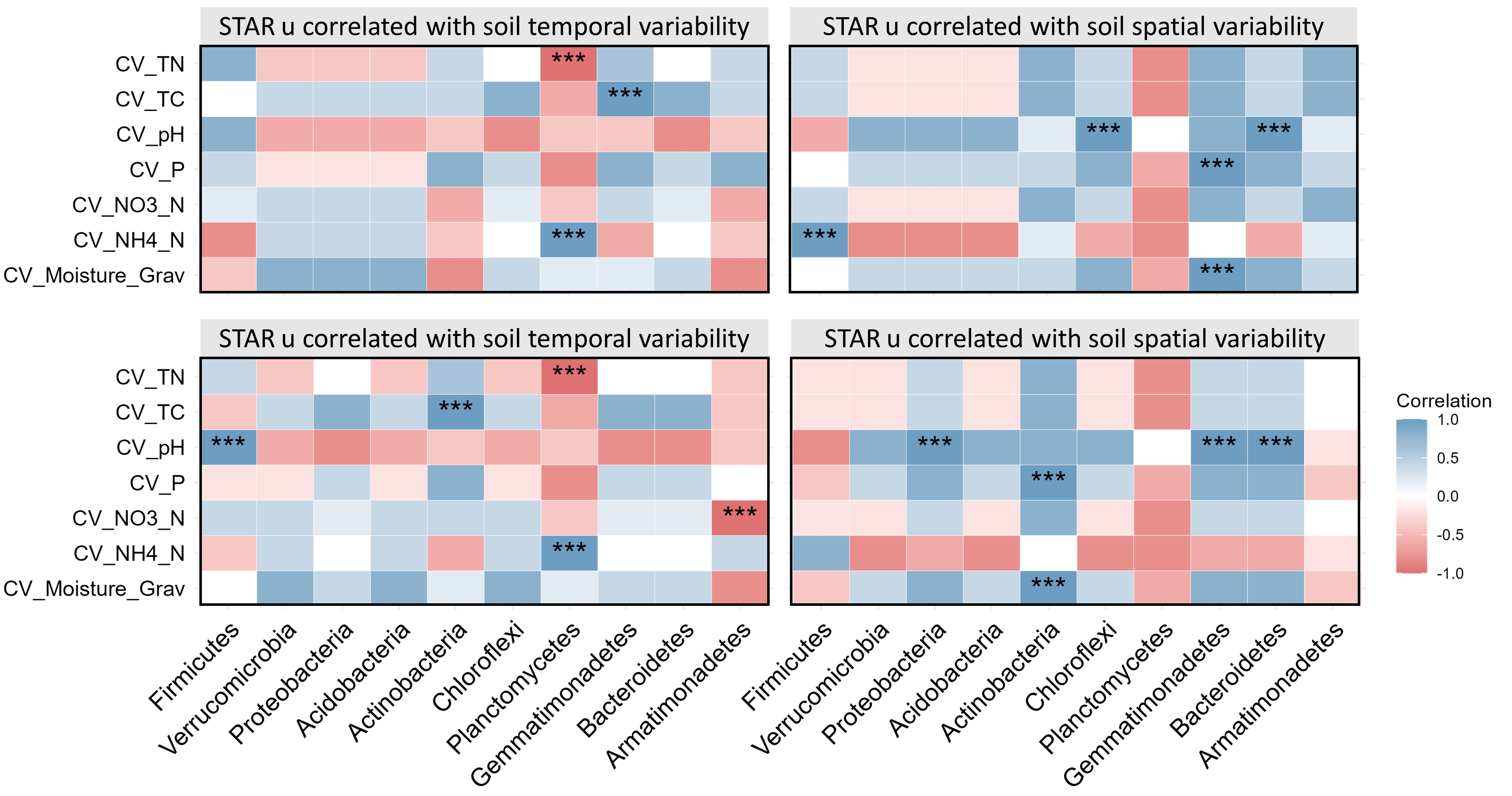


**Figure S15 The correlation between the variabilities of soil properties and the time-space interaction terms of different bacterial phyla.** The variability (coefficient of variation; CV) was treated as the soil heterogeneity. The correlation was examined as the Spearman correlation method. *** p < 0.001; ** p < 0.010; * p < 0.050.


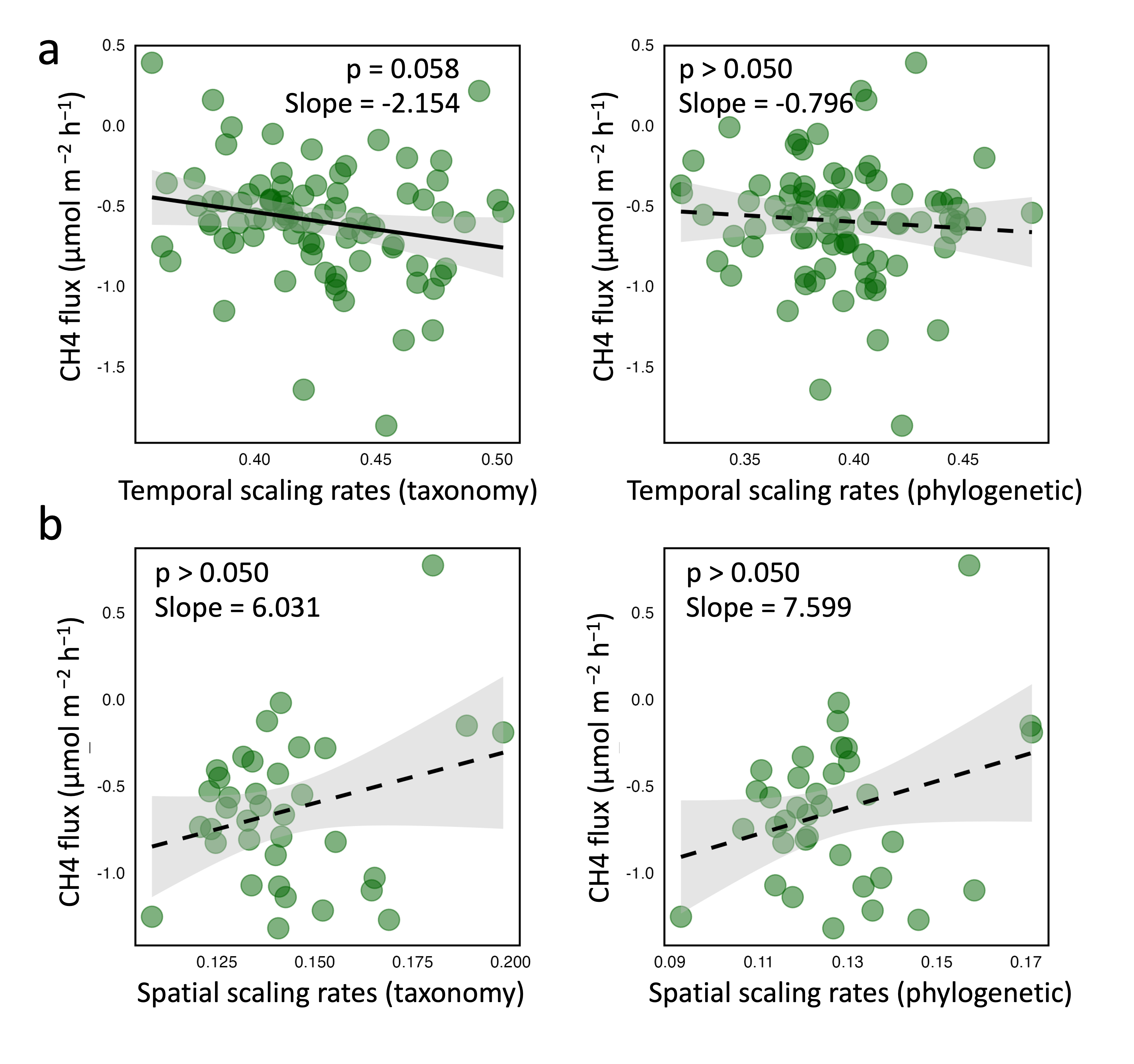


**Figure S16 Linear fitting between Spatiotemporal scaling rates and soil CH_4_ flux. a**, The correlation between temporal scaling rate and soil CH_4_ flux. **b**, The correlation between spatial scaling rate and soil CH_4_ flux. The significance was tested by the linear mixed model.

**
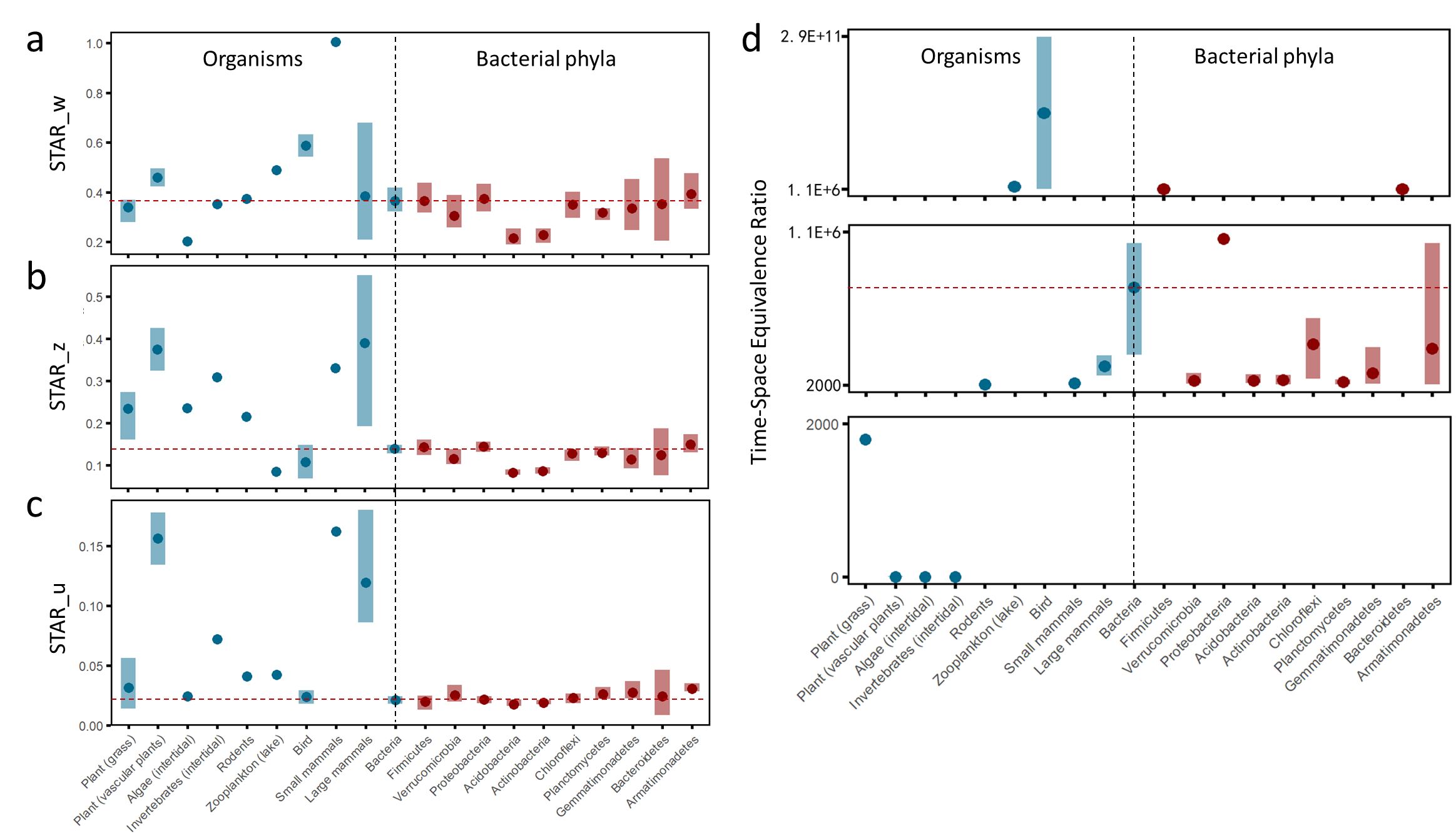
**

**Figure S17 Comparison of STAR exponents in micro- and macroorganisms.** **a-c**, Comparison of STAR w- (**a**), z- (**b**), and u- (**c**) values in micro- and macroorganisms. Recent data for micro/macroorganism communities were included in the analyses (a total of 61 pieces of data). The values of STAR spatiotemporal scaling rates of different bacterial phyla were all from our present study. The error bars indicate the maximum and minimum values.

**
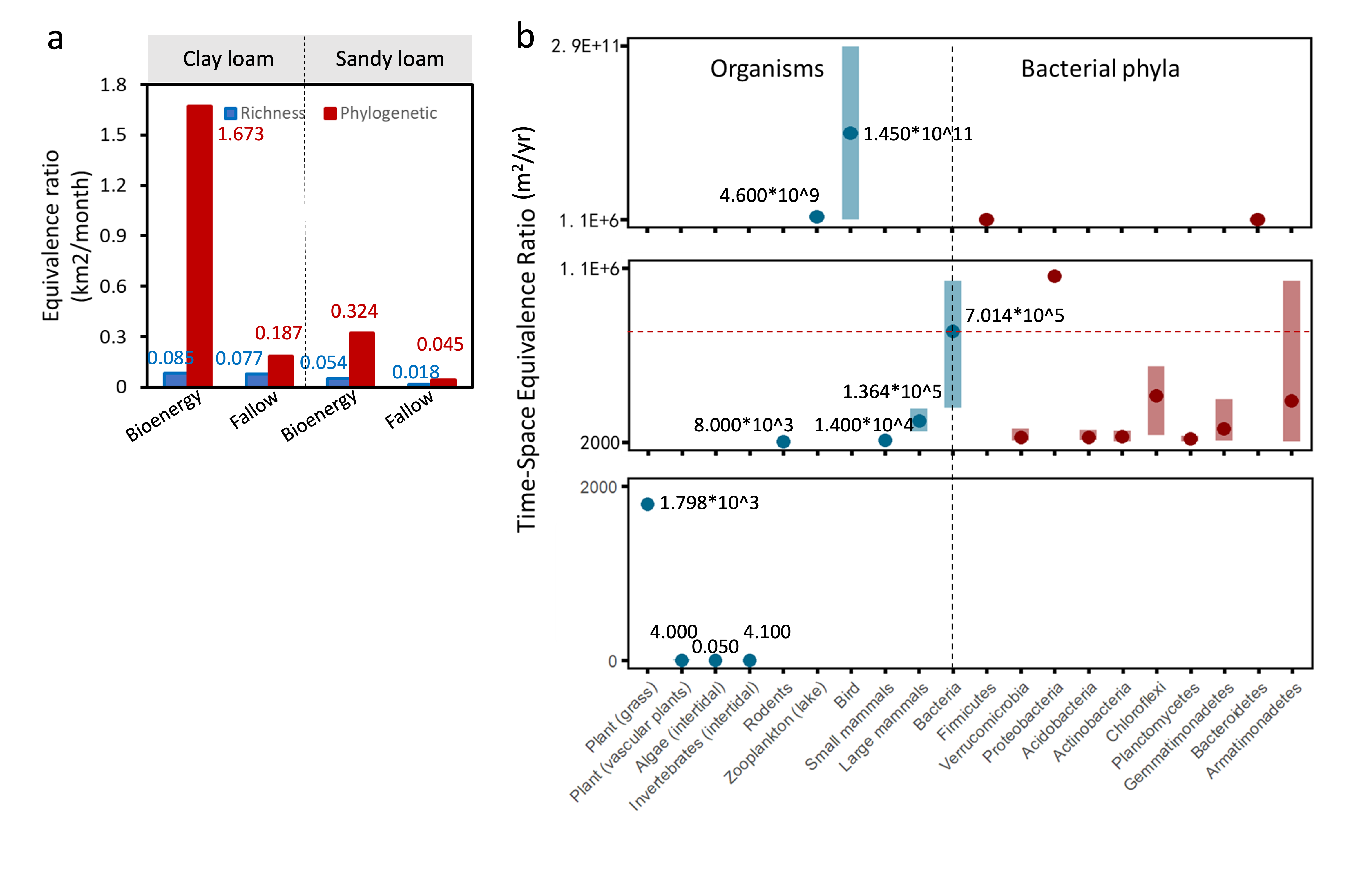
**

**Figure S18 The changes in bacterial space-time equivalence ratios among different treatments and the underlying mechanisms.** **a**, The richness-based and phylogenetic diversity-based bacterial space-time equivalence ratios in bioenergy cropping plots at sandy loam and clay loam sites. **b**, Comparison of space-time equivalence ratios in micro- and macroorganisms.


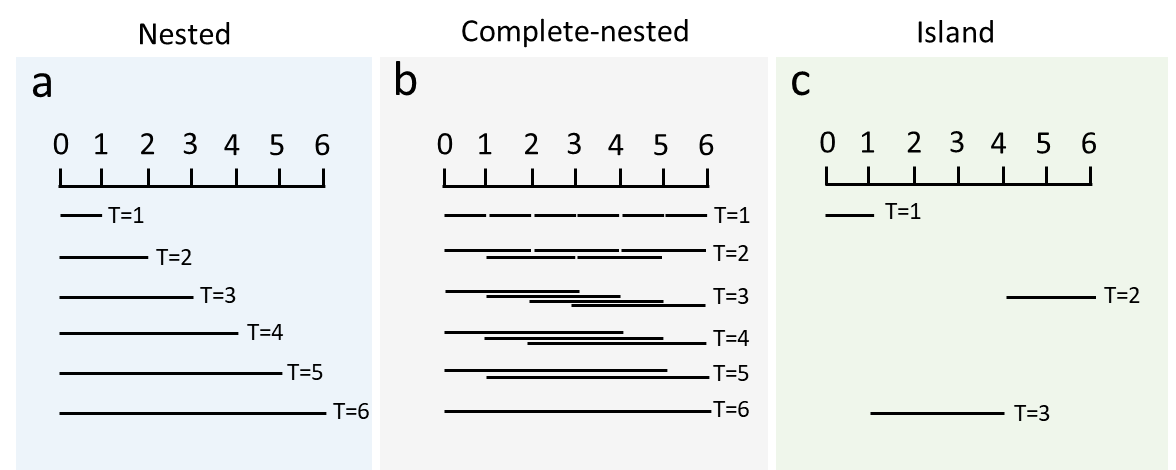


**Figure S19 Different methods for considering the time period when calculating the Species-Time relationship.**

**
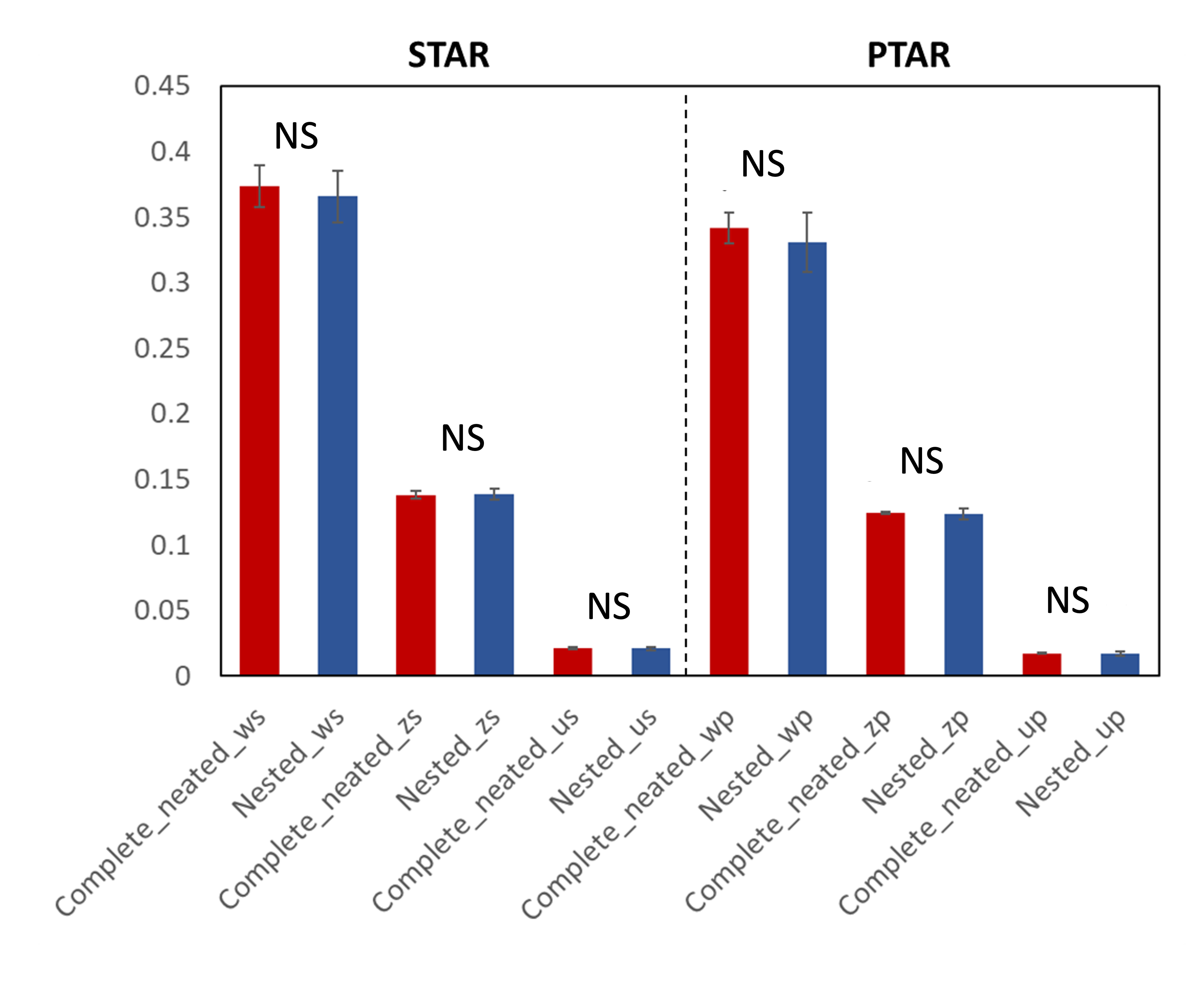
**

**Figure S20 The comparison of STAR and PTAR exponents between nested and complete nested methods.** The significance is examined by the *t*-test. NS: no significance (p > 0.050)

**Table S1 The spatiotemporal scaling rates of major common bacterial phyla.** SL: sandy loam soil; CL: clay loam soil; SG: switchgrass; FL: Fallow.

|  | **STAR w values** | |  |  | **STAR z values** | |  |  | **STAR u values** | |  |  |
| --- | --- | --- | --- | --- | --- | --- | --- | --- | --- | --- | --- | --- |
| phylum | SL_SG | SL_FL | CL_SG | CL_FL | SL_SG | SL_FL | CL_SG | CL_FL | SL_SG | SL_FL | CL_SG | CL_FL |
| Firmicutes | 0.438 | 0.350 | 0.356 | 0.317 | 0.161 | 0.123 | 0.158 | 0.125 | -0.022 | -0.012 | -0.024 | -0.017 |
| Verrucomicrobia | 0.257 | 0.297 | 0.276 | 0.389 | 0.101 | 0.118 | 0.102 | 0.137 | -0.021 | -0.026 | -0.019 | -0.033 |
| Proteobacteria | 0.322 | 0.371 | 0.365 | 0.434 | 0.131 | 0.147 | 0.140 | 0.154 | -0.018 | -0.023 | -0.018 | -0.024 |
| Acidobacteria | 0.190 | 0.196 | 0.220 | 0.254 | 0.076 | 0.081 | 0.079 | 0.090 | -0.016 | -0.016 | -0.016 | -0.021 |
| Actinobacteria | 0.222 | 0.197 | 0.254 | 0.242 | 0.080 | 0.084 | 0.094 | 0.085 | -0.017 | -0.018 | -0.020 | -0.017 |
| Chloroflexi | 0.297 | 0.321 | 0.381 | 0.400 | 0.110 | 0.128 | 0.136 | 0.133 | -0.018 | -0.023 | -0.023 | -0.026 |
| Planctomycetes | 0.334 | 0.335 | 0.288 | 0.314 | 0.127 | 0.143 | 0.122 | 0.123 | -0.027 | -0.031 | -0.021 | -0.023 |
| Gemmatimonadetes | 0.248 | 0.263 | 0.377 | 0.452 | 0.092 | 0.099 | 0.119 | 0.140 | -0.022 | -0.024 | -0.025 | -0.036 |
| Bacteroidetes | 0.206 | 0.307 | 0.361 | 0.536 | 0.075 | 0.127 | 0.105 | 0.187 | -0.008 | -0.025 | -0.016 | -0.046 |
| Armatimonadetes | 0.333 | 0.336 | 0.429 | 0.477 | 0.130 | 0.138 | 0.173 | 0.156 | -0.028 | -0.030 | -0.035 | -0.028 |
|  | **PTAR w values** | |  |  | **PTAR z values** | |  |  | **PTAR u values** | |  |  |
| phylum | SL_SG | SL_FL | CL_SG | CL_FL | SL_SG | SL_FL | CL_SG | CL_FL | SL_SG | SL_FL | CL_SG | CL_FL |
| Firmicutes | 0.375 | 0.272 | 0.317 | 0.280 | 0.142 | 0.102 | 0.125 | 0.096 | -0.018 | -0.006 | -0.017 | -0.009 |
| Verrucomicrobia | 0.198 | 0.232 | 0.191 | 0.288 | 0.082 | 0.099 | 0.072 | 0.108 | -0.013 | -0.018 | -0.008 | -0.022 |
| Proteobacteria | 0.273 | 0.319 | 0.326 | 0.411 | 0.111 | 0.131 | 0.123 | 0.142 | -0.012 | -0.018 | -0.014 | -0.024 |
| Acidobacteria | 0.151 | 0.163 | 0.197 | 0.206 | 0.060 | 0.065 | 0.060 | 0.067 | -0.010 | -0.010 | -0.006 | -0.013 |
| Actinobacteria | 0.187 | 0.159 | 0.232 | 0.208 | 0.062 | 0.064 | 0.077 | 0.077 | -0.011 | -0.011 | -0.012 | -0.013 |
| Chloroflexi | 0.198 | 0.213 | 0.255 | 0.280 | 0.074 | 0.088 | 0.084 | 0.087 | -0.010 | -0.014 | -0.009 | -0.015 |
| Planctomycetes | 0.233 | 0.228 | 0.214 | 0.237 | 0.088 | 0.100 | 0.086 | 0.093 | -0.017 | -0.020 | -0.012 | -0.016 |
| Gemmatimonadetes | 0.150 | 0.184 | 0.265 | 0.290 | 0.057 | 0.067 | 0.075 | 0.087 | -0.011 | -0.016 | -0.013 | -0.020 |
| Bacteroidetes | 0.149 | 0.230 | 0.257 | 0.384 | 0.052 | 0.097 | 0.064 | 0.134 | -0.002 | -0.015 | -0.004 | -0.027 |
| Armatimonadetes | 0.198 | 0.214 | 0.270 | 0.263 | 0.076 | 0.093 | 0.105 | 0.079 | -0.015 | -0.020 | -0.019 | -0.008 |

**Table S2 The spatial and temporal scaling rates of major common bacterial phyla.** The SAR and PAR were generated for each time point, while the STR and PTR were generated for each sampling position. Bold indicates significant differences, which were examined by the Turkey HSD test. SL: sandy loam soil; CL: clay loam soil; SG: switchgrass; FL: Fallow.

|  | **SAR_z** | | | | **PAR_z** | | | | **STR_w** | | | | **PTR_w** | | | |
| --- | --- | --- | --- | --- | --- | --- | --- | --- | --- | --- | --- | --- | --- | --- | --- | --- |
| Phylum | SL_FL | SL_SG | CL_FL | CL_SG | SL_FL | SL_SG | CL_FL | CL_SG | SL_FL | SL_SG | CL_FL | CL_SG | SL_FL | SL_SG | CL_FL | CL_SG |
| Acidobacteria | **0.083** | **0.072** | 0.096 | 0.087 | **0.068** | **0.058** | 0.075 | 0.069 | 0.268 | 0.267 | **0.305** | **0.266** | 0.201 | 0.201 | 0.233 | 0.223 |
| Actinobacteria | 0.085 | 0.080 | 0.085 | 0.089 | **0.070** | **0.066** | 0.072 | 0.074 | **0.272** | **0.326** | 0.296 | 0.297 | 0.211 | 0.212 | 0.242 | 0.239 |
| Armatimonadetes | 0.143 | 0.146 | 0.165 | 0.159 | 0.084 | 0.084 | 0.095 | 0.090 | **0.495** | **0.454** | **0.637** | **0.528** | 0.262 | 0.268 | **0.324** | **0.284** |
| Bacteroidetes | 0.119 | 0.110 | 0.139 | 0.143 | 0.087 | 0.083 | 0.101 | 0.099 | **0.394** | **0.294** | **0.605** | **0.525** | **0.272** | **0.297** | **0.409** | **0.338** |
| Chloroflexi | **0.125** | **0.114** | 0.136 | 0.144 | **0.082** | **0.075** | 0.091 | 0.098 | **0.407** | **0.385** | **0.467** | **0.442** | **0.253** | **0.240** | 0.317 | 0.312 |
| Firmicutes | 0.138 | 0.141 | **0.138** | **0.149** | 0.116 | 0.117 | 0.111 | 0.118 | **0.386** | **0.454** | **0.363** | **0.382** | **0.333** | **0.383** | **0.299** | **0.339** |
| Gemmatimonadetes | **0.096** | **0.088** | 0.131 | 0.134 | **0.062** | **0.055** | 0.080 | 0.082 | **0.373** | **0.347** | **0.530** | **0.466** | **0.225** | **0.182** | **0.317** | **0.277** |
| Planctomycetes | 0.146 | 0.138 | 0.144 | 0.133 | **0.104** | **0.095** | 0.106 | 0.097 | 0.457 | 0.446 | **0.426** | **0.400** | 0.310 | 0.312 | **0.300** | **0.337** |
| Proteobacteria | 0.145 | 0.143 | 0.150 | 0.146 | 0.132 | 0.13 | 0.132 | 0.131 | **0.440** | **0.406** | **0.469** | **0.407** | **0.375** | **0.410** | **0.419** | **0.397** |
| Verrucomicrobia | 0.105 | 0.105 | **0.131** | **0.109** | 0.086 | 0.083 | **0.098** | **0.081** | **0.382** | **0.318** | **0.465** | **0.371** | **0.277** | **0.262** | **0.331** | **0.290** |

**Table S3 The effects of bioenergy cropping (Switchgrass) on STAR and PTAR exponents, calculated by ln-response ratio.**

|  |  | **Total effect size** | | **Clay loam** | | **Sandy loam** | |
| --- | --- | --- | --- | --- | --- | --- | --- |
|  | **Exponents** | **ln-response ratio** | **variance** | **ln-response ratio** | **variance** | **ln-response ratio** | **variance** |
| STAR | w | -0.038 | 0.000 | -0.040 | 0.000 | -0.035 | 0.000 |
|  | z | -0.040 | 0.001 | -0.041 | 0.000 | -0.038 | 0.000 |
|  | u | -0.104 | 0.001 | -0.099 | 0.004 | -0.110 | 0.000 |
| PTAR | w | -0.0219 | 0.000 | -0.024 | 0.000 | -0.018 | 0.000 |
|  | z | -0.016 | 0.001 | -0.025 | 0.000 | -0.007 | 0.000 |
|  | u | -0.068 | 0.001 | -0.073 | 0.004 | -0.062 | 0.001 |

**Table S4 The effects of bioenergy cropping (Switchgrass) and soil types on soil properties.** Examined by a linear mixed model. SL: sandy loam soil; CL: clay loam soil; SG: switchgrass; FL: Fallow.

|  | Effect of SG at the SL site | | Effect of SG at the CL site | | Effect of CL in SG plots | | Effect of CL in FL plots | |
| --- | --- | --- | --- | --- | --- | --- | --- | --- |
|  | β | p | β | p | β | p | β | p |
| pH | 0.166 | <0.001 | -0.031 | 0.476 | -1.017 | <0.001 | -0.820 | <0.001 |
| Moisture | 0.126 | <0.010 | -0.027 | 0.729 | 0.296 | <0.001 | 0.495 | <0.001 |
| NO_3__N | -0.130 | <0.001 | 0.274 | <0.050 | 0.899 | <0.001 | 0.494 | <0.001 |
| NH_4__N | 0.068 | <0.050 | -0.072 | <0.050 | 0.122 | <0.010 | 0.261 | <0.001 |
| P | 0.747 | <0.001 | -0.193 | <0.001 | -2.219 | <0.001 | -1.279 | <0.001 |
| TN | 0.224 | <0.001 | 0.040 | 0.568 | 1.607 | <0.001 | 1.791 | <0.001 |
| TC | 0.303 | <0.001 | -0.019 | 0.773 | 1.433 | <0.001 | 1.755 | <0.001 |

**Table S5 The space-time equivalence ratios calculated for different treatments with different units.** SL: sandy loam soil; CL: clay loam soil; SG: switchgrass; FL: Fallow.

|  | **z** | **w** | **u** | **Model R^2^** | **Ratios of scales of equivalence (km^2^ /month)** | **Ratios of scales of equivalence (km^2^ /yr)** |
| --- | --- | --- | --- | --- | --- | --- |
| **Richness based** |  |  |  |  |  |  |
| SL_FL | 0.141 | 0.357 | -0.022 | 0.991 | 0.018 | 0.216 |
| SL_SG | 0.127 | 0.323 | -0.018 | 0.996 | 0.054 | 0.648 |
| CL_FL | 0.148 | 0.418 | -0.024 | 0.986 | 0.077 | 0.926 |
| CL_SG | 0.138 | 0.365 | -0.020 | 0.993 | 0.085 | 1.020 |
| **Phylogenetic based** |  |  |  |  |  |  |
| SL_FL | 0.127 | 0.320 | -0.018 | 0.998 | 0.045 | 0.540 |
| SL_SG | 0.114 | 0.279 | -0.013 | 0.995 | 0.324 | 3.888 |
| CL_FL | 0.134 | 0.389 | -0.021 | 0.984 | 0.187 | 2.244 |
| CL_SG | 0.119 | 0.334 | -0.015 | 0.993 | 1.673 | 20.076 |

**Table S6 Results of the Mantel test analysis for the bacterial community composition.** SL: sandy loam soil; CL: clay loam soil; SG: switchgrass; FL: Fallow. Bold represents a significance less than 0.050.

| Type | Soil property | r | | p | Type | Soil property | r | p |
| --- | --- | --- | --- | --- | --- | --- | --- | --- |
| SL_SG | **pH** | | **0.192** | **0.001** | CL_SG | **pH** | **0.114** | **0.009** |
|  | **Moisture** | | **0.180** | **0.001** |  | **Moisture** | **0.239** | **0.001** |
|  | **NO3_N** | | **0.161** | **0.001** |  | **NO3_N** | **0.115** | **0.017** |
|  | NH4_N | | 0.010 | 0.406 |  | **NH4_N** | **0.070** | **0.027** |
|  | **P** | | **0.096** | **0.003** |  | **P** | **0.145** | **0.004** |
|  | **TN** | | **0.121** | **0.003** |  | **TN** | **0.075** | **0.037** |
|  | **TC** | | **0.193** | **0.001** |  | TC | -0.005 | 0.508 |
| SL_FL | **pH** | | **0.190** | **0.001** | CL_FL | **pH** | **0.184** | **0.002** |
|  | **Moisture** | | **0.072** | **0.043** |  | Moisture | -0.022 | 0.673 |
|  | NO3_N | | -0.067 | 0.947 |  | NO3_N | -0.031 | 0.702 |
|  | **NH4_N** | | **0.103** | **0.014** |  | NH4_N | -0.025 | 0.713 |
|  | P | | 0.038 | 0.222 |  | **P** | **0.111** | **0.028** |
|  | TN | | 0.001 | 0.488 |  | TN | -0.017 | 0.646 |
|  | TC | | 0.017 | 0.338 |  | **TC** | **0.219** | **0.001** |

**References**

[1] V. Devictor, D. Mouillot, et al., “Spatial mismatch and congruence between taxonomic, phylogenetic and functional diversity: the need for integrative conservation strategies in a changing world,” *Ecology Letters* *13* (2010): 1030. <https://doi.org/10.1111/j.1461-0248.2010.01493.x>.

[2] D. S. Srivastava, M. W. Cadotte, et al., “Phylogenetic diversity and the functioning of ecosystems,” *Ecology Letters* *15* (2012): 637. <https://doi.org/10.1111/j.1461-0248.2012.01795.x>.

[3] T. Bell, D. Ager, et al., “Larger islands house more bacterial taxa,” *Science* *308* (2005): 1884. <https://doi.org/10.1126/science.1111318>.

[4] D. W. Rivett, S. B. Mombrikotb, et al., “Bacterial communities in larger islands have reduced temporal turnover,” *The ISME Journal* *15* (2021): 2947. <https://doi.org/10.1038/s41396-021-00976-0>.

[5] M. C. Horner-Devine, M. Lage, et al., “A taxa-area relationship for bacteria,” *Nature* *432* (2004): 750. <https://doi.org/10.1038/nature03073>.

[6] X. Guo, X. Zhou, et al., “Climate warming accelerates temporal scaling of grassland soil microbial biodiversity,” *Nature Ecology & Evolution* *3* (2019): 612. <https://doi.org/10.1038/s41559-019-0848-8>.

[7] Y. Wang, G. Lu, et al., “Meadow degradation increases spatial turnover rates of the fungal community through both niche selection and dispersal limitation,” *Science of the Total Environment* *798* (2021): 149362. <https://doi.org/10.1016/j.scitotenv.2021.149362>.

[8] S.-p. Li, P. Wang, et al., “Island biogeography of soil bacteria and fungi: similar patterns, but different mechanisms,” *Isme Journal* *14* (2020): 1886. <https://doi.org/10.1038/s41396-020-0657-8>.

[9] S. Woodcock, T. P. Curtis, et al., “Taxa-area relationships for microbes: the unsampled and the unseen,” *Ecology Letters* *9* (2006): 805. <https://doi.org/10.1111/j.1461-0248.2006.00929.x>.

[10] S. Carey, A. Ostling, et al., “Impact of curve construction and community dynamics on the species-time relationship,” *Ecology* *88* (2007): 2145. <https://doi.org/10.1890/06-1889.1>.

[11] A. J. Redford, N. Fierer, “Bacterial Succession on the Leaf Surface: A Novel System for Studying Successional Dynamics,” *Microbial Ecology* *58* (2009): 189. <https://doi.org/10.1007/s00248-009-9495-y>.

[12] G. F. Wells, H.-D. Park, et al., “Fine-scale bacterial community dynamics and the taxa-time relationship within a full-scale activated sludge bioreactor,” *Water Research* *45* (2011): 5476. <https://doi.org/10.1016/j.watres.2011.08.006>.

[13] A. Shade, J. G. Caporaso, et al., “A meta-analysis of changes in bacterial and archaeal communities with time,” *Isme Journal* *7* (2013): 1493. <https://doi.org/10.1038/ismej.2013.54>.

[14] H. Li, S. Yang, et al., “Temperature sensitivity of SOM decomposition is linked with a K-selected microbial community,” *Global Change Biology* *27* (2021): 2763. <https://doi.org/10.1111/gcb.15593>.

[15] T. Dai, D. Wen, et al., “Nutrient supply controls the linkage between species abundance and ecological interactions in marine bacterial communities,” *Nature Communications* *13* (2022): 175. <https://doi.org/10.1038/s41467-021-27857-6>.

[16] Y. Dou, J. Liao, et al., “Importance of soil labile organic carbon fractions in shaping microbial community after vegetation restoration,” *CATENA* *220* (2023): 106707. <https://doi.org/https://doi.org/10.1016/j.catena.2022.106707>.

[17] J. Zhou, S. Kang, et al., “Spatial scaling of functional gene diversity across various microbial taxa,” *Proceedings of the National Academy of Sciences* *105* (2008): 7768. <https://doi.org/doi:10.1073/pnas.0709016105>.

[18] B. Zhang, K. Xue, et al., “Power law in species-area relationship overestimates bacterial diversity in grassland soils at larger scales,” *Global Ecology and Biogeography* *33* (2024): e13825. <https://doi.org/10.1111/geb.13825>.

[19] R. Hestrin, M. R. Lee, et al., “The Switchgrass Microbiome: A Review of Structure, Function, and Taxonomic Distribution,” *Phytobiomes Journal* *5* (2021): 14. <https://doi.org/10.1094/pbiomes-04-20-0029-fi>.
